# EGCG inhibits the inflammation and senescence inducing properties of MDA-MB-231 triple-negative breast cancer (TNBC) cells-derived extracellular vesicles in human adipose-derived mesenchymal stem cells

**DOI:** 10.1186/s12935-023-03087-2

**Published:** 2023-10-13

**Authors:** Narjara Gonzalez Suarez, Yuniel Fernandez-Marrero, Mathieu P. A. Hébert, Marie-Eve Roy, Luc H. Boudreau, Borhane Annabi

**Affiliations:** 1 Laboratoire d’Oncologie Moléculaire, Département de Chimie, Université du Québec À Montréal and CERMO-FC, C.P. 8888, Succ. Centre-Ville, Montreal, QC H3C 3P8 Canada; 2 Cell Biology Department, NuChem Sciences, Montreal, QC H4R 2N6 Canada; 3 Department of Chemistry and Biochemistry, Université de Moncton and New Brunswick Center for Precision Medicine, Moncton, NB Canada

**Keywords:** EGCG, Triple-negative breast cancer, Extracellular vesicles, Adipose-derived mesenchymal stem cells, Inflammation, Senescence

## Abstract

**Background:**

Triple-negative breast cancer (TNBC) cells’ secretome can induce a pro-inflammatory phenotype in human adipose-derived mesenchymal stem cells (hADMSC). This can be prevented by the green tea polyphenol epigallocatechin-3-gallate (EGCG). The impact of EGCG on the paracrine regulation that the extracellular vesicles (EVs) specifically exert within the TNBC secretome remains unknown.

**Methods:**

EVs were obtained from a TNBC-derived serum-starved MDA-MB-231 cell model treated or not with EGCG under normoxic or hypoxic (< 1% O_2_) culture conditions. RNA-Seq analysis was used to assess the EVs’ genetic content. The modulation of inflammatory and senescence markers in hADMSC was evaluated by RT-qPCR using cDNA arrays and validated by immunoblotting. A protein profiler phospho-kinase array was used to explore signaling pathways.

**Results:**

While hypoxic culture conditions did not significantly alter the genetic content of MDA-MB-231-secreted EVs, the addition of EGCG significantly modified EVs genetic material at low oxygen tension. Gene expression of cancer-associated adipocyte pro-inflammatory markers *CXCL8*, *CCL2* and *IL-1β* was increased in hADMSC treated with EVs. Concomitantly, EVs isolated from MDA-MB-231 treated with EGCG (EGCG-EVs) downregulated *CCL2* and *IL-1β,* while inducing higher expression of *CXCL8* and *IL-6* levels. EVs activated CHK-2, c-Jun, AKT and GSK-3β signaling pathways in hADMSC, whereas EGCG-EVs specifically reduced the latter two as well as the serum starvation-induced senescence markers p21 and β-galactosidase. Finally, the mitochondrial content within the TNBC cells-derived EVs was found reduced upon EGCG treatment.

**Conclusion:**

This proof of concept study demonstrates that the chemopreventive properties of diet-derived polyphenols may efficiently target the paracrine regulation that TNBC cells could exert upon their surrounding adipose tissue microenvironment.

**Supplementary Information:**

The online version contains supplementary material available at 10.1186/s12935-023-03087-2.

## Significance statement

The present study reveals that diet-mediated intervention, such as through circulating EGCG, can alter the genetic material found within the triple-negative breast cancer (TNBC) cells-derived extracellular vesicles (EVs). EGCG further reduced the capacity of the EVs to trigger the pro-inflammatory and senescence processes which often are associated to a chemoresistance phenotype. EGCG caused a reduction in the mitochondrial content of cancer cells-derived EVs, reinforcing its overall antitumoral role. Circulating diet-derived polyphenols may therefore represent an efficient chemopreventive strategy to reduce the paracrine regulation that TNBC cells exert within their surrounding adipose tissue environment.

**Supplementary Information:**

The online version contains supplementary material available at 10.1186/s12935-023-03087-2.

## Background

The release of extracellular vesicles (EVs) loaded with proteins, lipids, and nucleic acids is an efficient mechanism for cell-to-cell communication [1]. EVs differ in size and origin, but they are all delimited by a phospholipid bilayer. Exosomes is the most accepted term for smaller EVs with a diameter between 30 and 150 nm and from endosomal origin [2]. On the other hand, microvesicles (also termed microparticles or ectosomes) represent a more heterogeneous population of particles that originate from the plasmatic membrane budding with a size ranging from 100 to 1000 nm [3]. Specific RNA, DNA and proteins can be sorted through these secreted vesicles and regulate the gene expression in cells from distant tissues or within the metastatic niche [3, 4]. Several studies have ascribed a role for EVs in mediating processes like inflammation [5–8] and tumor progression [4]. For instance, glioma-derived EVs harbouring the mutated variant III of the epidermal growth factor receptor (EGFRvIII) transferred this oncoprotein to other cancer cells and promoted the expansion of a more aggressive tumor phenotype [9].

Nowadays, it is well-accepted that tumors control and pre-condition the metastatic niche in part through the release of EVs [10]. However, little is known about the EVs-mediated paracrine regulation of cells within the tumor tissue microenvironment and how the neighbouring resident cells, particularly those from the adipose tissue, can be impacted in response to triple-negative breast cancer (TNBC) cells secretome. In this regard, it has been reported that tumor-derived EVs can trigger normal fibroblasts differentiation into cancer-associated fibroblasts (CAF) [11], induce immune suppression throughout the release of immune checkpoints [12, 13], and promote tumor metastasis [14].

Many factors can influence the molecular signature and composition of cell secretome and must be considered during in vitro studies using cell cultures. These include low oxygen hypoxic culture conditions, which mimic conditions found within solid tumours and in which cancer cells must molecularly adapt to survive [15]. The effect of hypoxia on the release mechanisms of EVs has been reviewed for different cell types [16], as well as its impact on the EVs’ cargo [15]. The main focus has been on the content and identity of the miRNAs (small non-coding RNAs) profile within the hypoxic-EVs due to their capacity to regulate gene expression [17]. In addition, EVs have been reported to carry and deliver functional mitochondria, free mitochondrial DNA (mtDNA) and their components with a regulatory impact on inflammation [8, 18, 19]. Consequently, the horizontal transfer of mitochondrial components between cells can modulate the recipient cell’s phenotype, including cell respiration [20] and cell viability [21, 22], contributing to a more resistant phenotype of the recipient cell [23, 24]. While studies in this topic have mostly been carried out on platelets [18] or neutrophils [25], only few have investigated the role of tumor-derived EVs packed with mitochondrial components. Sansone et al. reported that mtDNA transferred from EVs leads to the exit from dormancy of therapy-induced cancer stem-like cells, inducing resistance to hormone therapy in metastatic breast cancer patients [26].

Our previous research demonstrated that the secretome content of a TNBC-derived cell line triggered cell migration and the acquisition of a pro-inflammatory phenotype in a human mesenchymal stem cell line derived from the adipose tissue (hADMSC) [27]. Furthermore, acquisition of such inflammatory phenotype was prevented by the diet-derived polyphenol epigallocatechin-3-gallate (EGCG) [27]. EGCG has well-known antitumoral and antioxidant effects [28]. More recently, it has been reported in a murine model that EGCG inhibited the exosome-mediated infiltration of tumor-associated macrophages (TAM) by transferring miR-16 [29]. Given breast adipose tissue-derived mesenchymal stromal/stem cells are crucial components proned to respond to cues from the tumor microenvironment, and a key step initially involved in this process might be their de-differentiation into tumor supporting phenotypes [30], blocking their response within the tumor microenvironment could therefore serve as a novel chemopreventive strategy effective against breast cancer cell paracrine regulation.

In the present study, we aimed to evaluate the contribution of the tumoral-elicited EVs in the induction of a pro-inflammatory phenotype in hADMSC, and whether the genetic content of EVs isolated from TNBC-derived MDA-MB-231 cells treated with EGCG was altered. Given the recently discovered link between inflammation and senescence processes [31, 32], we addressed whether EVs’ can trigger senescence in hADMSC and whether this can be prevented by EGCG. Since the mitochondrial content could also contribute to the acquisition of such phenotype in hADMSC, we also analyzed the effect of EGCG on the sorting of mitochondria components within the EVs.

## Materials and methods

### Materials

Bovine serum albumin (BSA), sodium dodecyl-sulphate (SDS) and EGCG were obtained from Sigma-Aldrich Canada (Oakville, ON). Phosphate-buffered saline (PBS) buffer solution (pH ~ 7.4) was purchased from (Wisent, St-Bruno, QC). For the SDS–polyacrylamide gel electrophoresis (SDS-PAGE) as well as the enhanced chemiluminescence (ECL) reagents, they were from Bio-Rad (Mississauga, ON). Micro bicinchoninic acid protein assay reagents were purchased from Pierce (Rockford, IL). The antibodies against BIP, P16, IL-6, phospho-AKT, AKT, phospho-GSK3β, and GSK3β were obtained from Cell Signaling Technology Inc (Danvers, MA). The anti-Tubulin antibody was purchased from ICN Biomedical (Aurora, OH), anti-P21 was from Abcam (Cambridge, UK), and anti-CD9, CD63 and CD81 from ThermoFisher Scientific (Waltham, MA). Horseradish peroxidase-conjugated anti-rabbit and anti-mouse IgG secondary antibodies were from Jackson ImmunoResearch Laboratories (West Grove, PA).

### Cell culture and procedure to generate the conditioned media for EVs isolation

The human adipose-derived mesenchymal stem cells (hADMSC) and TNBC-derived cell line MDA-MB-231 were purchased from American Type Culture Collection (ATCC, Manassas, VA). hADMSC were grown in Mesenchymal Stem Cell Basal Medium (ATCC, PCS-500-030) and supplemented with Mesenchymal Stem Cell Growth Kit Low Serum (ATCC, PCS-500-040). Cells were used within 3–6 passages. They were further reported to be able to undergo adipogenesis [27]. MDA-MB-231 cells were grown in EMEM Medium (Wisent, 320-036-CL) supplemented with 10% fetal bovine serum (FBS) and used within 8 passages for extracellular vesicles (EVs) isolation. All cells were cultured at 37 °C under a humidified 95–5% (v*/*v) mixture of air and CO_2_. For EVs isolation, approximately 2–5 × 10^6^ of MDA-MB-231 cells were seeded in 175 cm^2^ cell culture flasks and cultured in EMEM (Wisent, 320-036-CL) supplemented with 10% FBS. When the cells reached approximately 70–80% confluence, the monolayer was washed twice with negative media (NM) and left in a 20 mL/flask of NM with or without 30 µM EGCG for 48 h. Then, the conditioned media was harvested and clarified by centrifugation at 1500 g for 20 min to remove cell debris and stored at 4 °C for a maximum of one week pending EVs isolation. For the experiments at different oxygen tensions, after the monolayers reached a 70% of cell confluency, cells were incubated for 48 h at 37 °C under normoxic (21% of O_2_ and 5% CO_2_) or in hypoxic (O_2_ ≤ 1% and 5% CO_2_) culture conditions. Next, RNA was extracted for genes expression analysis.

### Extracellular vesicles isolation

The clarified conditioned media was concentrated form 40 mL to 10 mL by centrifugation using Amicon Ultra-3 K (Millipore, Oakville, ON). EVs were then isolated using ExoQuick-TC Exosomes precipitation Solution Kit (SBI) following the manufacturer protocol. The exosomes-enriched EVs pellet was resuspended in 500 µL of PBS for dynamic light scattering (DLS) particle size analysis or in Trizol for total RNA isolation and subsequent RNA-Seq analysis. In the case of in vitro experiments, the vesicles pellet was resuspended in 200 µL of un-supplemented Mesenchymal Stem Cell Basal Medium (BM). Vesicles obtained under normoxia and in the presence of EGCG were termed as EGCG-EVs to distinguish them from those obtained without the catechin (EVs).

### Extracellular vesicles relative quantification

A portion of the EVs pellet was used for the relative quantification of the particles. Samples were incubated in the presence of membrane-specific dye MemGlow™ 488 (100 nM, Cytoskeleton Inc., Denver, CO) in a total volume of 100 µL containing 20 µL of the EVs pellet suspension, 20 min at room temperature and in the dark. Samples were then diluted with the addition of 100 µL of PBS and processed for flow cytometry analysis for total EVs count. Briefly, using the high-resolution flow cytometer Cytoflex (Beckman Coulter, Indianapolis, IN), the number of MemGlow-positive vesicles was quantified in an acquisition volume of 30 µL (gating strategy is shown in Additional file 6: Fig.S1). EVs count in the original sample was obtained using the following equation: number of particles/µL = (P2*6.7)/20.

### Dynamic light scattering

EVs size/diameter was assessed by DLS. The mean hydrodynamic diameter of EVs was calculated by fitting a Gaussian function to the measured size distribution. Prior to DLS measurements, each sample was centrifuged at 300 g for 10 s to pellet large aggregates; 50 μL of the sample was added to a ZEN00400 cuvette, and DLS measurements were conducted at 25 °C using a Nano ZSP Zetasizer (Malvern Instruments Ltd., UK) operating at 633 nm and recording the back-scattered light at an angle of 175°. The sample was allowed to equilibrate for 2 min before each measurement. DLS was recorded for 200 s with three replicate measurements. Signal intensity was transformed to volume distribution assuming a spherical shape of EVs and using the Malvern Instruments Ltd. software.

### Western blotting

hADMSC were lysed in a buffer containing 1 mM NaF, 1 mM Na_3_VO_4_ and a phosphatase inhibitory cocktail (END Millipore, Germany). Then, the proteins (10 µg) were separated under denaturing conditions using SDS-PAGE. Next, proteins were electro-transferred to polyvinylidene difluoride (PVDF) membranes and blocked with 5% non-fat dry milk in Tris-buffered saline (150 mM NaCl, 20 mM Tris–HCl, pH 7.5) at 0.3% Tween-20 (TBS-T; Bioshop, TWN510-500) for 1 h at room temperature. PVDF membranes were washed three times in TBS-T and incubated with the appropriate primary antibodies (1/1,000 dilution) overnight at 4 °C with agitation. All primary antibodies were resuspended in a solution of TBS containing 3% BSA and 0.1% sodium azide (Sigma-Aldrich). Finally, the PVDF membranes were incubated for 1 h with horseradish peroxidase-conjugated donkey anti-rabbit or anti-mouse IgG at 1/2500 diluted in TBS-T at 5% non-fat dry milk. ECL was used to visualize the immunoreactive material (Bio-Rad, Mississauga, ON). Tubulin detection within the hADMSC lysates was used as loading control. To confirm the presence of exosomes, the EVs were lysed in RIPA buffer 1X (NaCl 150 mM, NP-40 1%, SDS 0.1%, TRIS 50 mM, at pH 7.5), and 4 µg of protein were separated in a 12% SDS-PAGE under non-denaturing conditions. Next, the standard Western blot protocol was used to detect CD63, CD9 and CD81 exosome markers. A human phosphor-kinase array kit (Proteome Profiler™, R&D System® Inc, Minneapolis, MN) was used to screen the activated pathways in the hADMSC upon 1 h treatment with the EVs and EGCG-EVs. The detection was performed according to the manufacturer’s instructions. Densitometry analysis were performed using ImageJ software version 1.53e.

### Characterization of MDA-MB-231-derived EVs interaction with hADMSC

The EVs preparations were evaluated by flow cytometry for their capacity to interact with hADMSC. Once isolated, EVs were labelled using MemGlow™ 488 as described above, then washed with PBS by ultracentrifugation at 100,000 g for 1 h at 4 °C and resuspended in BM. hADMSC (10,000 cells/mL) were incubated with the EVs at a ratio of 1:1 (cells:EVs) in basal media and suspensions kept for 2 h at 37 °C. Next, the population of fluorescent cells was analyzed by flow cytometry using an Accury C6 device (Beckman Coulter, Indianapolis, IN).

### Confocal microscopy

To detect the presence of EVs within the hADMSC, 10^4^ cells were seeded in EBD plates (Fitchburg, Wisconsin, WI). The next day, growth media was collected, and 500 µL of BM supplemented or not with MemGlow-labelled EVs was added on cells and incubated for 2-h. Fluorescent vesicles attached to the cells were visualized using a live imaging confocal microscope (Nikon Instruments Inc., Melville, NY). The experiment was performed two times, and pictures of three different fields were taken. To determine whether the mitochondrial components present within the EVs could be efficiently transferred to hADMSC, MDAMB-231 cells were seeded and incubated over night with mitoTracker Deep Red (MTR) at a final concentration of 200 nM in negative media (NM). Then, the cell monolayer was washed with PBS and kept for 24 hours in NM, and EVs (MTR+EVs) were isolated as described in the Methods section and protected from light. hADMSC were seeded on top of tissue culture glass slides (Falcon, NY, USA) previously coated with Poly-lysine. 200 μl of MTR+EVs were then resuspended in NM and incubated for 4 hours. Cells were then labelled with 100 nM MemGlow, incubated for 20 minutes at RT and in the dark. Finally, cells were fixed in 1% paraformaldehyde solution and DAPI was added to stain the nucleus. Pictures were taken using a fluorescence microscope.

### Senescence detection assay

The level of β-galactosidase (β-gal) activity was assessed using the CellEvent™ Senescence Green Detection kit (ThermoFisher Scientific). hADMSC (8000 cells/well) were seeded onto a poly-L-lysine (Sigma Aldrich) pre-coated glass chamber (Nunc, Rochester, NY). Once the cells adhered, the growth media was removed, and either EVs, EGCG-EVs at ratio 1:2 (Cells:EVs) or BM were added for 48 h. Then, cells were washed and stained according to the manufacturer’s instructions for the detection of β-gal activity. The nucleus was stained with DAPI. Experiments were performed in duplicate for each biological condition, and three pictures/well were taken. The results were presented as the percent of positive cells per field (number of β-gal positive cells/total of cells)*100.

### Chemotactic cell migration assay

The experiments performed to evaluate the effect of EVs on cell migration were carried out using the Real-Time Cell Analyzer (RTCA) Dual-Plate (DP) Instrument of the xCELLigence system (Roche Diagnostics, Mississauga, ON). The migration plates (CIM-Plates 16) have conventional trans-wells (8 µm pore size) with gold electrode arrays on the upper chamber to record real-time cell migration. Before seeding the cells, the wells of the upper chamber were coated with 25 µL of 0.15% gelatin in PBS and incubated for one hour at 37 °C, followed by a wash with PBS. Then, hADMSC (10,000 cells/well) were added and co-cultured with the EVs at a ratio of 1:1 (cells:EVs) in the upper chamber of the device. The RTCA-DP Instrument software measured the impedance values and expressed them in arbitrary units as Normalized Cell Migration Index. Chemotaxis in response to BM supplemented with 1% FBS and added at the bottom chamber was monitored for 8 h.

### Quantification of the mitochondria-containing vesicles

The cell culture media supernatant was filtered using a 1.2 µm syringe filter to remove any remaining cell contaminants. This is a well validated approach to evaluate clinical samples without performing ultracentrifugation [33] For each sample, 5 µL of the supernatant was combined with 1 µL of anti-CD44-FITC (Biolegend), 1 µL of MitoTracker Deep Red (100 nM final concentration, ThermoFisher Scientific) and 93 µL of 0.2 µm filtered PBS. Labeling was performed by incubating the mixture for 15 min at room temperature in the dark. Samples were then processed on the Attune NxT flow cytometer (ThermoFisher Scientific) for quantification of the EVs subpopulation as previously validated [18, 34, 35]. Gating strategies were established using a non-relevant anti-CD41-FITC antibody (Biolegend, San Diego, CA) for the MDA-MB-231-derived EVs. EVs subpopulation profile (particles size of ≥ 200 nm) was determined by quantification of the CD44^+^/MitoTracker Deep Red^−^ events (MPs) and the CD44^+^/MitoTracker Deep Red^+^ events (mitoMPs).

### Citrate synthase activity assay

The citrate synthase activity was performed as previously described [36]. Briefly, imidazole (Sigma-Aldrich) was resuspended in water at 6.8 mg/mL (pH 8.0). The reaction medium was then prepared by adding 0.1 mM of DTNB (5,5-dithio-bis-(2-nitrobenzoic acid)) sodium salt (Sigma-Aldrich) and 0.1 mM of AcetylCoA (Sigma-Aldrich) to the imidazole solution. The oxaloacetate solution was prepared by dissolving oxaloacetic acid in imidazole buffer at 0.2 mg/mL. The cell culture media supernatant (100 μL) was centrifuged at 17,800 g for 90 min with the resulting pellet resuspended in 5 µL of imidazole solution. In a 96-well plate, 200 µL of the reaction medium, 20 µL of the oxaloacetate solution and 5 µL of sample were subsequently added to the wells. The plate was then placed in a Biotek Synergy H1 Hybrid Microplate Reader where the absorbance was measured at 412 nm for 4 min at 30-s intervals (9 total reads) with continuous plate shaking between reads. The slopes (∆A/min) obtained from the reads were used to calculate the activity (AE; U/mL). For consideration, the DTNB has an extinction coefficient (ε) of 13.6 mL/(cm*µmol), the total volume is 225 µL, the sample volume is 5 µL and the light path is 0.643 cm. Each well of the 96-well plate has a diameter (d or 2 × radius (r)) of 6.675 mm.

### Total RNA isolation, cDNA synthesis, and RT^2^ Profiler™ PCR arrays

Total RNA was extracted from cell monolayers using 1 mL of TRIzol reagent for a maximum of 3 × 10^6^ cells as recommended by the manufacturer (Life Technologies, Gaithersburg, MD). For cDNA synthesis, 1 µg of total RNA was reverse transcribed using the R2 First Strand kit (QIAGEN, Valencia, CA). The cDNA was stored at − 80 °C prior to PCR. To detect the genes modulated upon the EVs and the EGCG-EVs treatment, the hADMSC (100,000 cells/well) were seeded in a six-well plate (SARSTEDT, Montreal, QC). The next day the growth media was removed, and the cells were co-incubated with BM or with the EVs or EGCG-EVs resuspended in BM and at a ratio of 1:0.5 (cells:EVs) for twenty-four hours at 37 °C and 5% of CO_2_. Then, cells were resuspended in Trizol for RNA isolation. The RT^2^ Profiler™ PCR Array for Human Inflammatory Cytokines and Receptors (PAHS-181Z) and Human Cellular Senescence (PAHS-050ZD) were used according to the manufacturer’s protocol (QIAGEN). The detailed list of the genes assessed can be found at the manufacturer’s website (https://geneglobe.qiagen.com/us/product-groups/rt2-profiler-pcr-arrays). Using real-time quantitative PCR, we analyzed the expression of a panel of genes related to the inflammatory response and senescence markers. Relative gene expression was calculated using the 2^∆∆C^_T_ method (“delta-delta” method), in which C_T_ indicates the fractional cycle number where the fluorescent signal crosses the background threshold. This method normalizes the ∆∆C_T_ value of each sample using five housekeeping genes (B2M, HPRT1, RPL13A, GAPDH, and ACTB). The normalized fold change (FC) values are then presented as average FC = 2 (average ∆∆C_T_). Only genes amplified less than 35 cycles were analyzed. The resulting raw data were then analyzed using the PCR Array Data Analysis Template (http://www.sabiosciences.com/pcrarraydataanalysis.php). This integrated web-based software package automatically performs all ∆∆C_T_ -based FC calculations from the uploaded raw threshold cycle data.

### Total RNA library preparation and sequencing

The isolated vesicles preparations were resuspended in 500 µL of Trizol in triplicate per condition for library preparation. Total RNA was isolated using Trizol (ThermoFisher) and RNeasy mini kit (Qiagen) according to the manufacturer’s instructions. RNA was quantified using Qubit (ThermoFisher Scientific), and RNA quality control was assessed with the Bioanalyzer RNA 6000 Nano assay on the 2100 Bioanalyzer system (Agilent Technologies, Mississauga, ON). Transcriptome libraries were generated using the KAPA mRNA-Seq HyperPrep kit (Roche) using a poly-A selection (ThermoFisher Scientific). **S**equencing was performed on the Illumina NextSeq500, obtaining around 20 M single-end 75 bp reads per sample.

### Reads alignment and differential expression analysis

Reads were 30 trimmed for quality and adapter sequences using the Trimmomatic (version 0.35). Only reads with at least 50 bp length were kept for further analyses. Trimmed reads were aligned to the reference human genome version GRCh38 (gene annotation from Gencode version 37, based on Ensembl 103) using STAR version 2.7.1a [37]. Gene expressions were obtained as read count directly from STAR and computed using RNA-Seq by Expectation Maximization (RSEM) [38] to get normalized gene and transcript level expression, in TPM values, for these stranded RNA libraries. Differential expression analysis was performed using DESeq2 version 1.22.2 [39]. The package limma [40] was used to normalize expression data and read counts data were analyzed using DESeq2. Principal component analysis (PCA) for the first two most significant components was conducted with R packages [41] found in iDEP (integrated Differential Expression and Pathway) analysis [42] was also used to determine significant differentially expressed genes (DEG) with DESeq2 false discovery rate (FDR) adjusted p-value of 0.05 and fold-change with a cut-off of two. Gene expression was scaled and centered across samples using the mean and standard deviation and *k*-means clustering was performed using the consensus of least 10-independent runs using the R package *ComplexHeatmap* [43]. Pathways enrichment analysis was performed with selected genes using the *PathfindR* package [44]. Gene ontology (GO)-SLIM PANTHER 7.0 (protein annotation through evolutionary relationship) analysis of biological process and protein class were performed in the online platform (Gene Ontology Resource), using the Homo sapiens database as reference list.

### Statistical data analysis

Unless otherwise stated, data was expressed as mean ± standard error of the mean (SEM) from three or more independent experiments. Hypothesis testing was conducted using the Mann–Whitney test (two-group comparisons) or Wilcoxon signed-rank tests for independent or paired samples respectively. For multiple comparisons, the hypothesis was testing using Kruskal–Wallis, followed by a Dunn post-test. Critical values below 0.05 were deemed statistically significant and accordingly denoted in the figures (*: *p* < 0.05, **: *p* < 0.01). All statistical analyses were performed using the GraphPad Prism 8.0.1 software (San Diego, CA).

## Results

### *Characterization of the MDA-MB-231-derived EVs and validation of the *in vitro* approach.*

**Fig. 1:**
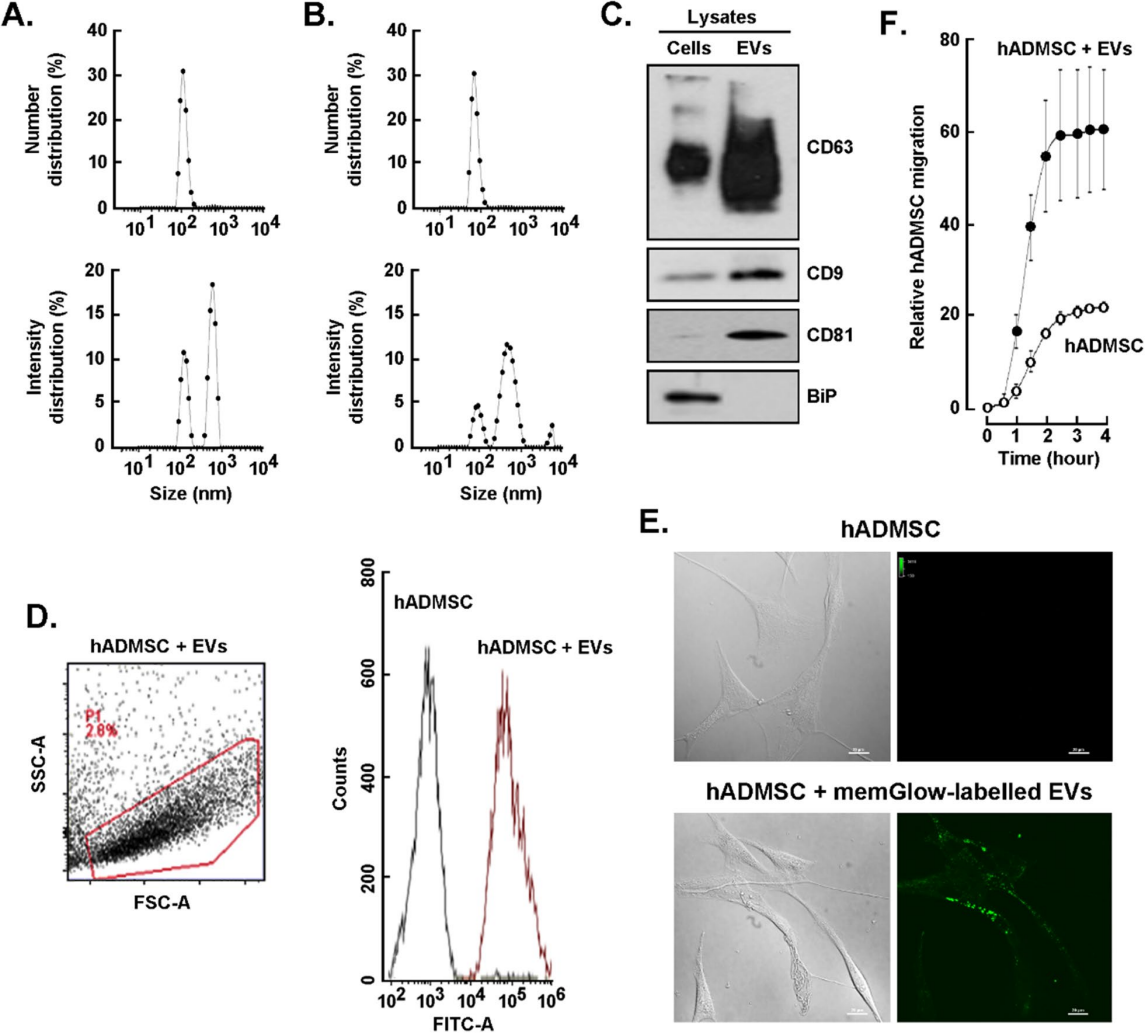
*Characterization of the EVs isolated from the MDA-MB-231 cells conditioned media*. Serum-starved triple-negative breast cancer-derived MDA-MB-231 cells were cultured for 48 h in the absence or presence of 30 µM EGCG. Conditioned media was next collected, concentrated, and extracellular vesicles (EVs) isolated as described in the Methods section. Dynamic light scattering particle size analysis of the **A** EVs and **B** EGCG-EVs distribution of the particles by number (upper panels) and by intensity (lower panels) of the refracted light. A representative experiment out of four is presented. **C** Immunoblotting of the exosomes enriched proteins CD9, CD63 and CD81, and of the negative marker BIP in MDA-MB-231 cell lysate and EVs lysate. **D** Gating strategy of the flow cytometry experiment performed to assess MemGlow-488 labelled EVs interaction with hADMSC. Merged histogram was obtained by the measurement by flow cytometry of the untreated cells (black lines) and the cells incubated with stained-EVs (red lines). A representative experiment out of two is presented. **E** Representative microscopy images of hADMSC incubated for two hours with the MDA-MB-231 cells-derived EVs labelled with MemGlow-488. A representative experiment out of three is presented (scale bar is 20 µm). **F** Relative cell migration rate of hADMSC treated with EVs (closed circle) or basal media (BM, open circle) in response to basal media supplemented with 1% FBS. Migration experiments were performed three times in quadruplicate. *Diameter in nanometers* (d.nm), *polydispersity index* (Pdl), intensity weighted mean hydrodynamic size of the particles (Z-Average). Statistically significant differences were determined by the non-parametric comparison test Mann–Whitney, * p < 0.05

To characterize the EVs release, serum-starved TNBC-derived MDA-MB-231 cells were cultured in the presence or absence of 30 µM EGCG for 48 h. A concentration documented to not alter MDA-MB-231 cell viability [45–47]. Conditioned media was next collected, and EVs were isolated as described in the Methods section. Particle distribution of both EVs preparations were analyzed using DLS. EVs samples showed a single peak with a mean diameter of ~ 100 nm, which corresponds with the expected exosome size, whether isolated from control cells (Fig. 1A, upper panel) or EGCG-treated cells (Fig. 1B, upper panel). However, when the peaks were analyzed as percent of intensity, we detected the presence of an additional population with sizes bigger than those expected for exosomes (Fig. 1A, B, lower panels). This suggests that samples are heterogeneous and this is also reflected by the polydispersity index which approximated 1. Nevertheless, the expression of the exosome enriched proteins CD9, CD63 and CD81 was confirmed by immunoblotting in the EVs lysates (Fig. 1C), using BiP as a protein not expected to be enriched within this fraction since it rather associates with secretory pathways [48]. Hence, one may safely consider our samples as a mixture of particles with different origins and will be referred to from now on as EVs rather than exosomes.

Next, EVs’ capacity to interact with the hADMSC and their impact on the recipient cell’s behaviour was investigated. EVs were labelled using MemGlow, an amphiphilic probe with high specificity for the plasmatic membrane and incubated for 2 h with hADMSC. Flow cytometry analysis confirmed that fluorescent EVs highly interacted (≥ 99%) with hADMSC (Fig. 1D). In addition, confocal fluorescent microscopy further demonstrated the presence of fluorescent EVs associated with hADMSC (Fig. 1E). Finally, hADMSC were treated with the EVs at a 1:1 ratio (cell:EVs) and cell migration assessed in response to basal media supplemented with 1% FBS. hADMSC treated with EVs had a 3–fourfold increase in cell migration rate in comparison to untreated cells (Fig. 1F). These results demonstrate that the MDA-MB-231 derived-EVs can trigger a pro-migratory adaptive response in hADMSC, confirming a potential bidirectional chemoattractive movement from adipose tissue cells towards the tumor microenvironment.

### Transcriptomic analysis and impact of EGCG on the EVs load released by MDA-MB-231 cells.

**Fig. 2:**
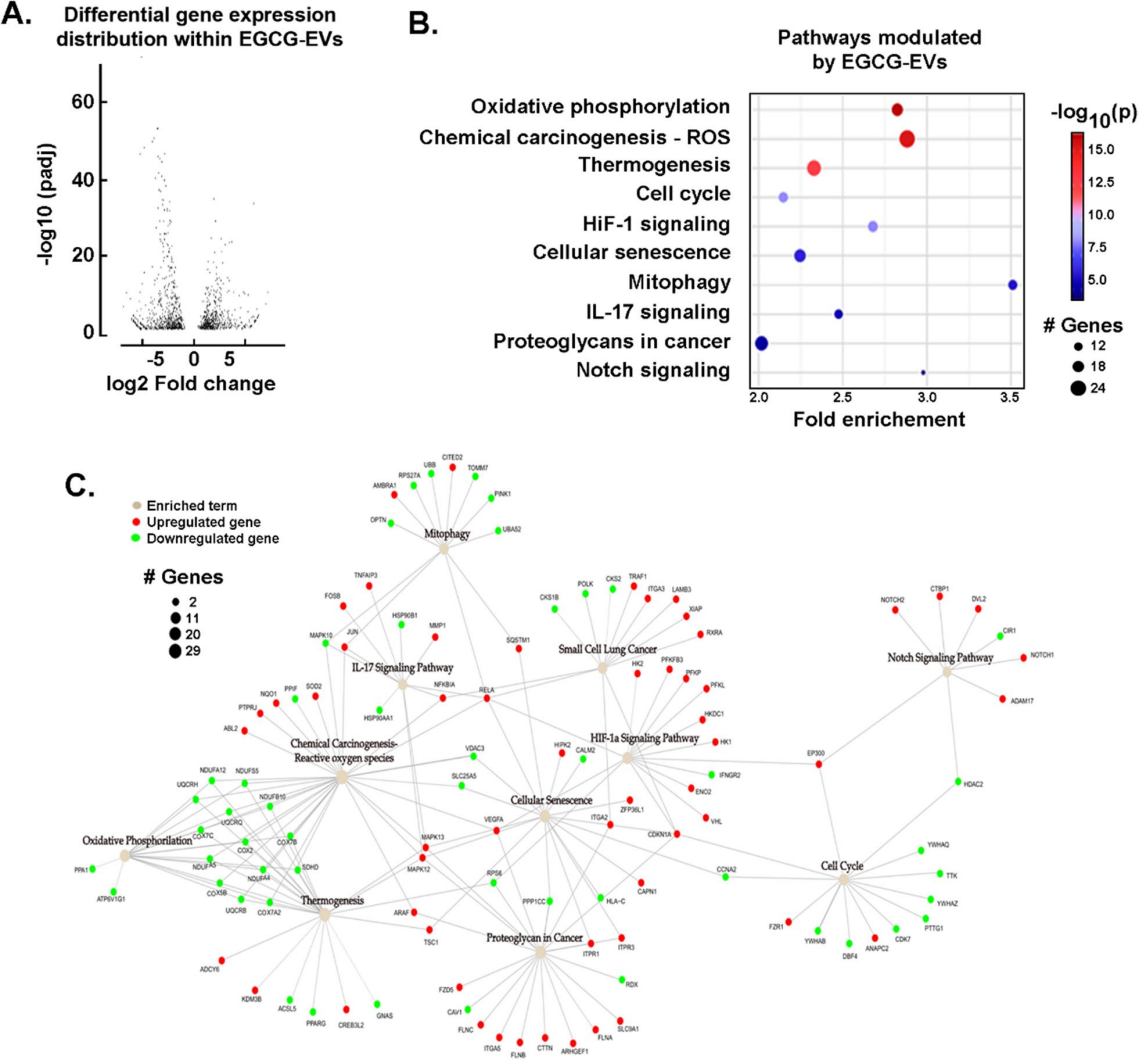
*Modulation of the EVs cargo by EGCG*. The extracellular vesicles (EVs) were isolated from serum-starved MDA-MB-231 cells treated or not with 30 μM EGCG for 48 h. **A** Volcano plot for the expression profile of differentially expressed genes (DEG) with an adjusted *p*-value < 0.05 was selected as the threshold. **B** KEGG pathways enrichment analysis resulted from the comparison of DEG (EGCG-EVs vs EVs) with absolute fold change (FC) > 2, and adjusted *p*-value < 0.05. **C** Network graph showing the enriched pathways and their respective genes. Enriched terms are coloured in beige, while upregulated and downregulated genes are coloured in red and green, respectively

MDA-MB-231-EVs are heterogeneous, and many factors can influence their content. Next, we investigated the specific transcript content packaged into the EVs following EGCG treatment. Our RNA-Seq analysis identified a total of 1116 differentially expressed genes (DEG) between EVs isolated from control (EVs) or EGCG-treated cells (EGCG-EVs) (Fig. 2A, an EXCEL sheet table is provided as Additional file 1). An asymmetric DEG distribution was observed with a slight tendency to gene downregulation in EGCG-EVs (619 downregulated vs 497 upregulated). Next, we searched for pathways enrichment containing the identified DEG using an active subnetwork-based algorithm [44]. Among the pathways enriched, cellular senescence, cell cycle, signaling pathways associated with IL-17, HIF-1 and Notch were observed (Fig. 2B). Interestingly, the highest genes induced in EGCG-EVs were found to associate with mitochondria-related pathways, and further included oxidative phosphorylation, chemical carcinogenesis-reactive oxygen species, and mitophagy (Fig. 2B). The latter further interconnected with thermogenesis, proteoglycans in cancer and small cell lung cancer biomarkers (Fig. 2C).

### TNBC cell-derived EVs trigger specific signaling pathways in hADMSC.

To address whether both MDA-MB-231-derived EVs’ preparations activated different downstream signaling cascades in hADMSC, we first quantified the EVs by flow cytometry as described in the Methods section (Additional file 6: Fig. S1A–C). Importantly, no statistical difference in the mean of fluorescence intensity (MFI) between the MemGlow-labelled EVs and EGCG-EVs was observed (Additional file 6: Fig. S1D), and neither in their interaction capacity with hADMSC (Additional file 6: Fig. S2). Interestingly, in terms of number of particles, EGCG-treated MDA-MB-231 were found to release more EVs than untreated cells (Additional file 6: Fig. S1E).

**Fig. 3:**
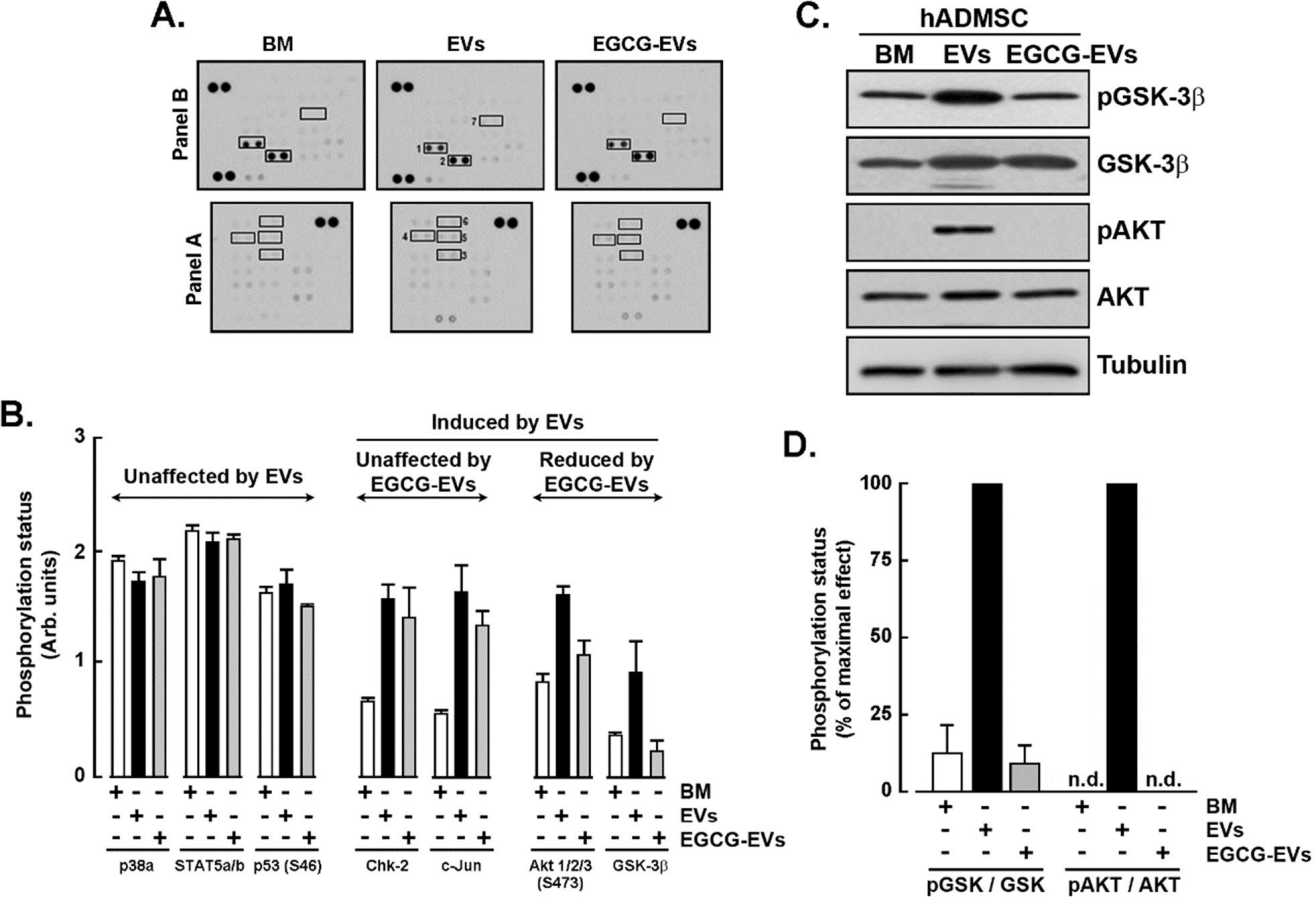
*Signalling cascades triggered by the EVs*. The hADMSC were incubated for one hour in basal media (BM), EVs, or EGCG-EVs using a Cell:EVs ratio of 1:0,5 (#:#). Cells were next lysed as described in the Methods section for Western blotting analysis. A phospho-kinase array was used to detect pathways activation states. **A** Immunoblotting results (1, p38a; 2, STAT5a/b; 3, p53; 4, Chk-2; 5, c-Jun; 6, Akt 1/2/3; 7, GSK-3b), and **B** Densitometric analysis of the highlighted immunoreactive spots performed using the ImageJ software. **C** Validation of the phosphorylated and total states of GSK-3β (Ser9) and AKT (Ser473) by immunoblotting. A representative experiment out of two is presented. **D** Ratios of the phosphorylated/total forms of AKT and GSK-3β resulted from the densitometric analysis performed with ImageJ. Mitogen-activated protein kinases (p38); signal transducer and activator of transcription 5A/B (STAT5a/b); Checkpoint kinase-2 (Chk-2); c-Jun N-terminal kinases JNK (c-Jun); protein kinase B signaling pathway (AKT); glycogen synthase kinase-3 (GSK-3); tumor protein 53 (p53)

Next, downstream phosphorylated intermediates were evaluated with a phospho-kinase immunoblotting array in hADMSC lysates isolated upon incubation with basal media (BM), EVs, or EGCG-EVs (Fig. 3A). Densitometry analysis perfomed on duplicate lysates from two independent membranes demonstrated that P38a, STAT5a/b and P53 phosphorylation status were induced, but unmodified upon any treatment (Fig. 3B). Such screen further revealed that Checkpoint kinase 2 (CHK-2) and c-Jun N-terminal kinases JNK (c-Jun) phosphorylation were induced upon EVs and EGCG-EVs treatments (Fig. 3B). Interestingly, EGCG-EVs demonstrated an inhibitory effect on the protein kinase B signaling pathway (AKT) and the glycogen synthase kinase-3 beta (GSK-3β) (Fig. 3B). The latter two were further validated using individual antibodies against their phosphorylated and total protein states (Fig. 3C). Levels of phosphorylation status profiles confirmed the phospho-kinase array results (Fig. 3D), suggesting that EGCG altered the signalling pathway triggered by the MDA-MB-231-derived EVs and involved in cell survival and proliferation.

### MDA-MB-231-derived EVs trigger the induction of a pro-inflammatory phenotype in hADMSC.

**Fig. 4:**
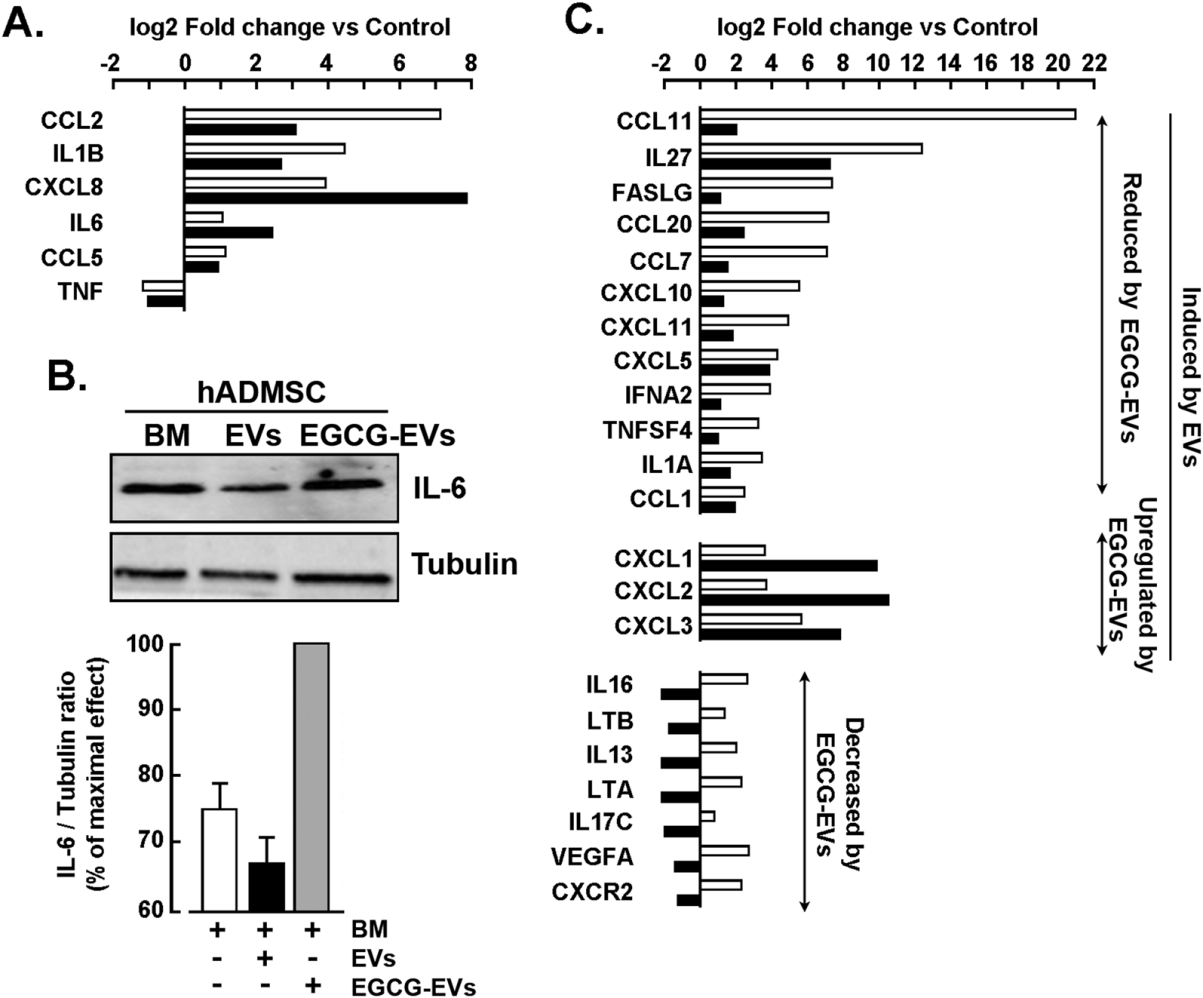
*Induction of a pro-inflammatory molecular signature by the MDA-MB-231 cells-derived EVs*. The hADMSC were incubated for 24 h in Basal Media (BM, Control), EVs (white bars) or EGCG-EVs (black bars) with a Cell: EVs ratio of 1:0,5. Next, total RNA was isolated, and cDNA was synthesized. Gene expression levels were determined by qPCR using a Human Inflammatory Cytokine and Receptors RT2-Profiler gene array kit. Densitometric analysis was performed using the ImageJ software.** A** The fold change (FC) expression of genes related to the cancer-associated adipocyte (CAA) phenotype. Validation of the arrays results for CAA genes was performed in two independent experiments. **B** Immunoblotting of interleukin-6 (IL-6) and tubulin (10 µg protein/well). Immunoblotting is representative of three experiments. **C** FC of selected genes from the array to highlight the modulatory effect of the EGCG-EVs

Induction of a pro-inflammatory phenotype by the TNBC secretome was previously reported in hADMSC, and this was prevented by EGCG [27]. Here, we assessed whether the different EVs isolated had an effect on the induction of a pro-inflammatory program within hADMSC. Then, cells were incubated for 24 h in serum-free media in the presence of EVs or EGCG-EVs, and total RNA was extracted from hADMSC and transcribed to cDNA. Levels of gene expression associated with inflammation were assessed by RT-qPCR using the Human Inflammatory Cytokines and Receptors RT^2^ Profiler gene array. A cut-off of a fold change (FC) greater or equal to two was defined. Genes related to the cancer-associated adipocytes (CAA) phenotype were found induced, and these included the C–C motif chemokine ligand 2 (*CCL2*), interleukin-1 beta (*IL-1B*), and C-X-C motif chemokine ligand 8 (*CXCL8*) (Fig. 4A). However, the C–C motif chemokine ligand 5 (*CCL5*) and the tumor necrosis factor (*TNF*) were not induced. Interestingly, the EGCG-EVs induced a higher upregulation of *CXCL8* and interleukin-6 (*IL-6*) (Fig. 4A), and the latter IL-6 expression was further confirmed at the protein level (Fig. 4B).

In addition to the induction of the CAA biomarkers, other pro-inflammatory genes were increased upon incubation with the EVs and included the C–C motif chemokine ligands 7, 11, 20 (*CCL7*, *CCL11*, *CCL20*), FAS ligand (*FASLG*), the C-X-C motif chemokine ligands 5 and 10 (*CXCL5*, *CXCL10*), and interleukin-27 (*IL-27*) (Fig. 4C). At the same time, EGCG-EVs reduced their expression, except for *CXCL5*, while they increased the expression of *CXCL1-3*. They also completely downregulated several interleukins (*IL-16*, *IL-13*, *IL-17C*), and other pro-inflammatory markers such as lymphotoxin-beta also known as tumor necrosis factor C (*LTB/TNF-C*), vascular endothelial growth factor A (*VEGFA*), and the C-X-C motif chemokine ligands 2 (*CXCR2*) (Fig. 4C).

### MDA-MB-231-derived EVs trigger hADMSC senescence

**Fig. 5:**
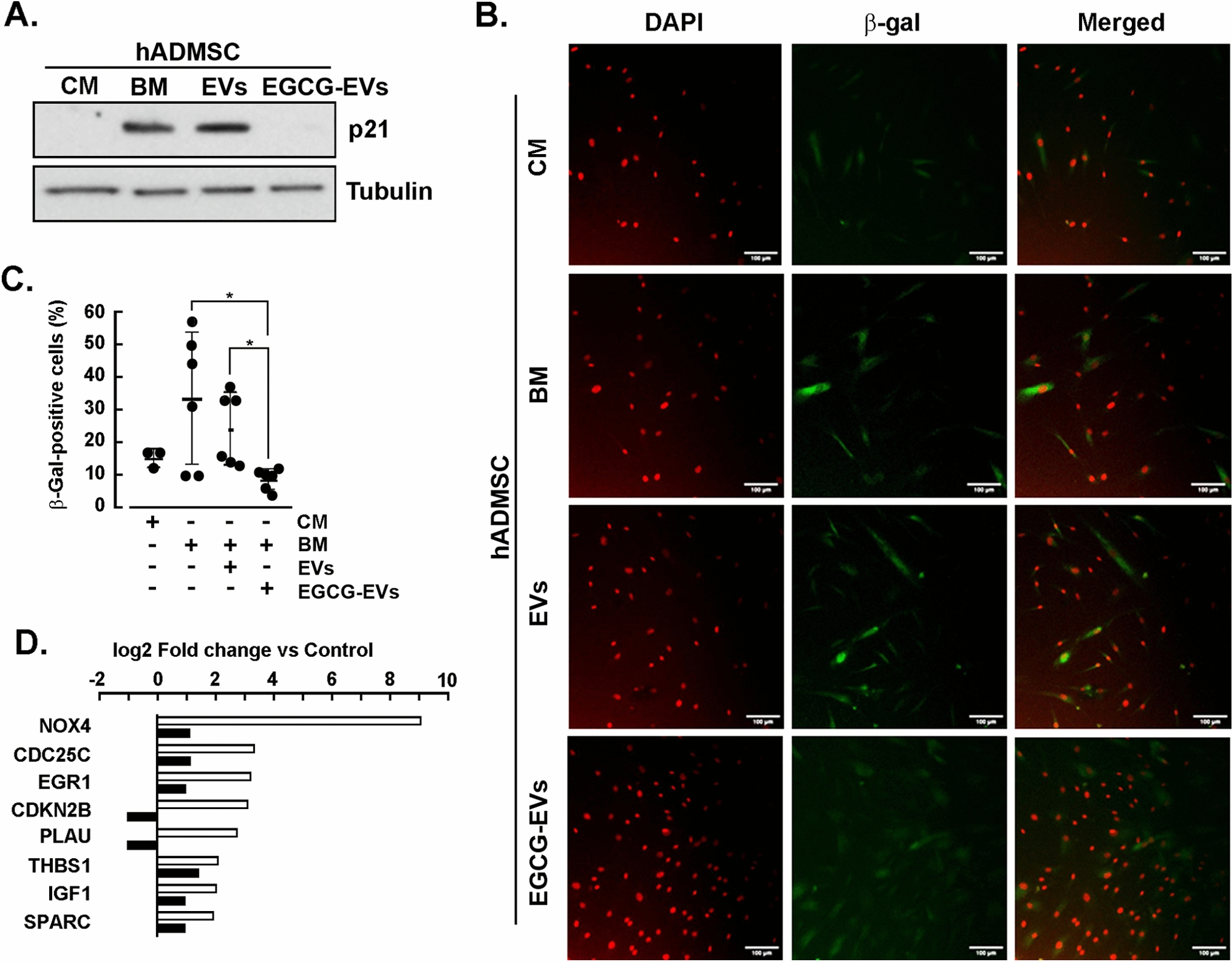
*EGCG-EVs rescue hADMSC from serum-starvation-induced senescence*. hADMSC were incubated for 24 h in complete media (CM), serum-deprived basal media (BM), EVs or EGCG-EVs at a ratio Cell:EVs of 1:0.5. hADMSC were collected for protein and total RNA as described in the Methods section. **A** Immunoblotting detection of the senescence biomarker p21 and of the loading control tubulin from control hADMSC lysates, treated with CM, BM, or the respective EVs. Immunoblotting is representative of three independent experiments. **B** Confocal microscopy of hADMSC treated for 48 h at a Cell: EVs ratio of 1:2. The nucleus was stained with DAPI (red), and the expression of the senescence-associated β-galactosidase (β-gal) marker is coloured in green. One out of three experiments is presented. **C** Histograms showing the percent of positive β-gal cells obtained upon 48 h of treatment. **D** Gene expression of other senescence markers modulated in hADMSC by EGCG-EVs (black bars) compared with the expression level of the genes in cells incubated with EVs (white bars), using as cut-off a log2 FC ≥ 2 and quantified by qPCR using the Human Senescence RT2-Profiler gene array kit. The percent of positive β-gal cells/field was calculated using the following equation: (number of positive cells /total of cells)*100. The Kruskall-Wallis test determined statistically significant differences, showing a * p < 0.05

Cancer-associated inflammation is one of the hallmarks of cellular senescence [31], and the link between both processes has been proposed as a mechanism for cancer chemoresistance. Since our experiments were performed without growth factors, we wished to assess the impact of EVs or EGCG-EVs on serum starvation-induced hADMSC senescence. Senescence was effectively found induced upon 24 h of serum starvation as P21 expression was triggered (BM condition) independently of the presence of EVs (Fig. 5A). However, EGCG-EVs treatment completely prevented serum-starvation induction of P21. This was further assessed at the cellular level through the expression of the primary senescence marker β-galactosidase (β-gal) [49]. Hence, hADMSC were incubated with EVs or EGCG-EVs at 1:2 cells/EVs ratio for 48 h, then washed and stained for the expression of β-gal as described in the Method section (Fig. 5B). In line with the increased expression of P21, the extent of β-gal positive cells increased with either BM or EVs-treated cells, and such increase was prevented upon EGCG-EVs treatment (Fig. 5C). Total RNA was extracted, cDNA synthetized and used to screen for senescence biomarkers expression with the Human Senescence RT^2^-Profiler RT-qPCR gene array. EVs treatment triggered NADPH oxidase 4 (*NOX4*), cell division cycle 25C (*CDC25C*), early growth response 1 (*EGR1*), cyclin-dependent kinase inhibitor 2B (*CDKN2B*), plasminogen activator urokinase (*PLAU*), thrombospondin 1 (*THBS1*), insulin-like growth factor 1 (*IGF1*) and the secreted protein acidic and rich in cysteine (*SPARC*) which were all significantly reduced in EGCG-EVs-treated hADMSC (Fig. 5D). The only gene increased by the EGCG-EVs, while not induced by the EVs, was the superoxide dismutase 2 (*SOD2*). Taking all these results together, it appears that the EGCG-EVs can rescue hADMSC from senescence induced upon serum starvation.

### Presence of mitochondria within MDA-MB-231-derived EVs

**Fig. 6:**
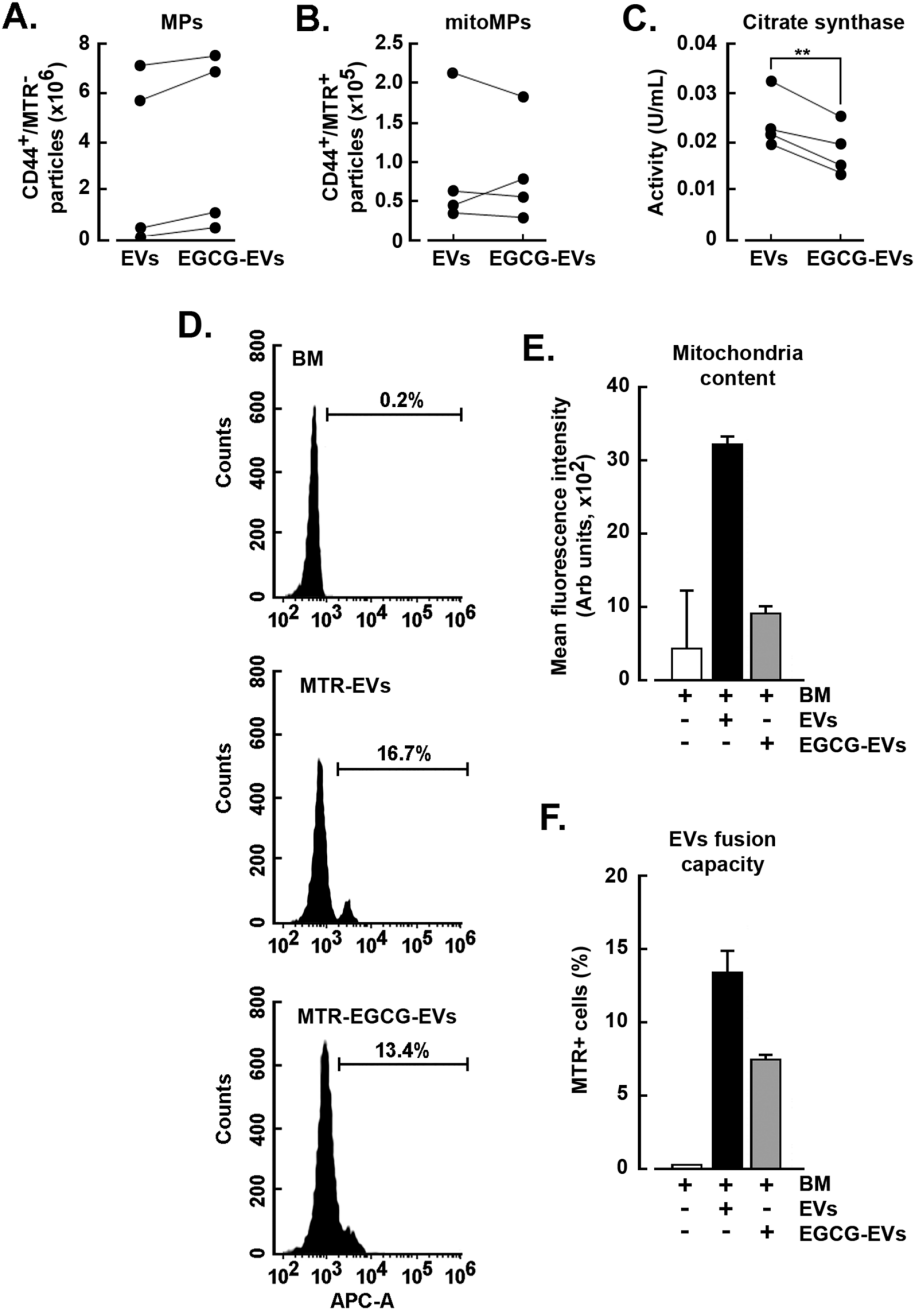
*EGCG reduces the mitochondrial content within the EVs*. Serum-starved MDA-MB-231 cells were cultured for 48 h in the presence or absence of 30 µM EGCG. EVs were isolated, stained with anti-CD44-FITC and MitoTracker Deep Red (MTR), and analyzed by flow cytometry. Four independent experiments were spaced in time from cell passage 3 to 8. The dots represent the mean of the counting, and results from the same experimental day were connected with a line in graphs A-C. Paired *t*-test was performed; **P < 0.01. **A** The total number of CD44^+^/MTR^−^ microparticles (MPs) detected in EVs or EGCG-EVs. **B** Quantification of the mitochondria-containing particles (CD44^+^/MTR^+^, mitoMPs) in the EVs or EGCG-EVs. Four independent experiments were performed. **C** Citrate synthase activity was measured in particles isolated from the conditioned media as described in the Methods section. **D** Dot plot resulting from the flow cytometry analysis detecting the presence of mitochondria delivered by the EVs in hADMSC after incubation with basal media (BM, negative control), mitoTracker-labelled EVs (MTR-EVs), or mitoTracker-labelled ECGC-EVs (MTR-EGCG-EVs). **E** Mean of the fluorescence intensity of the EVs or EGCG-EVs -delivered mitochondria within hADMSC. **F** The percent of hADMSC positive for the presence of mitochondria delivered by the EVs or EGCG-EVs

Significant expression of genes related to mitochondrial processes were highlighted during the RNA-Seq analysis performed in the genetic material within the EVs. Hence, we took a deeper look into those mitochondria-related genes and found that most of them were effectively detected in EVs and downregulated in the EGCG-EVs samples (Table 1). Consequently, we tracked mitochondrial content within isolated EVs and assessed whether EGCG treatment altered it. CD44 cell surface expression was used to quantify the total population of MDA-MB-231 derived EVs (MPs, Fig. 6A) while MitoTracker Deep Red (MTR) was used to identify mitochondrial material in the vesicles (mitoMPs, Fig. 6B). Although not statistically significant, EGCG-EVs had a tendency to higher MPs content, but a decrease in the mitoMPs subpopulation. Our flow cytometry results were supported by citrate synthase activity, which correlates with mitochondrial content, and in which the EGCG-EVs supernatant had less activity (Fig. 6C). Moreover, when hADMSC were incubated with the MTR-stained EVs or MTR-stained EGCG-EVs, 17% and 13% respectively of the cells became positive (Fig. 6D), whereas the MFI was reduced by more than half for those cells treated with the MTR-stained EGCG-EVs (Fig. 6E). The potential inhibitory effect of EGCG over the mitoTracker dye was ruled out since no variations in the MFI was detected in the MTR-stained MDA-MB-231 after being incubated at two different concentrations of the polyphenol (Additional file 6: Fig. S3). Moreover, uptake of mitoMPs within hADMSC and mitochondria delivery were observed confirming that the EVs cargo material can be taken up by the recipient cells (Additional file 6: Fig. S4; and refer to the 3D animation files provided as Additional file 2, Additional file 3, and Additional file 4). Altogether, these results suggest that EGCG causes a reduction in the EVs mitochondrial content that could eventually be transferred to the recipient cells upon EVs fusion (Fig. 6F).

**Table 1:** Fold change regulation of mitochondria-related genes detected in the EGCG-EVs vs EVs

Genes	log2 FC	*p*-adjusted value	Protein
MT-ATP8	− 6.64	3.99E-33	ATP synthase 8
MCUB	− 5.60	0.013846	Mitochondrial Calcium Uniporter Dominant Negative Subunit Beta. An integral component of the mitochondrial inner membrane
MT-ATP6	− 4.50	1.75E-11	ATP synthase 6
MT-ND2	− 4.33	4.73E-64	NADH dehydrogenase 2
MT-ND4	− 3.90	1.67E-44	NADH dehydrogenase 4
MT-CO2	− 3.81	8.09E-15	Cytochrome C oxidase II
MT-CYB	− 3.38	4.72E-12	Cytochrome b
MT-ND5	− 3.35	1.97E-34	NADH dehydrogenase 5
MT-CO3	− 3.16	4.84E-11	Cytochrome C oxidase III
MT-ND3	− 3.15	4.86E-44	NADH dehydrogenase 3
MT-ND4L	− 3.01	3.07E-38	NADH 4L dehydrogenase
MT-ND1	− 2.98	3.01E-29	NADH dehydrogenase 1
MT-CO1	− 2.41	9.83E-19	Cytochrome C oxidase I
MT-ND6	− 2.03	3.01E-10	NADH dehydrogenase 6
FIS1	− 2.03	0.0415208	Component of a mitochondrial complex that promotes mitochondrial fission
VDAC1	− 1.08	0.0247492	Voltage Dependent Anion Channel 1. A major component of the outer mitochondrial membrane

### Impact of EGCG and low oxygen tension on the sorted genes within the EVs

**Fig. 7:**
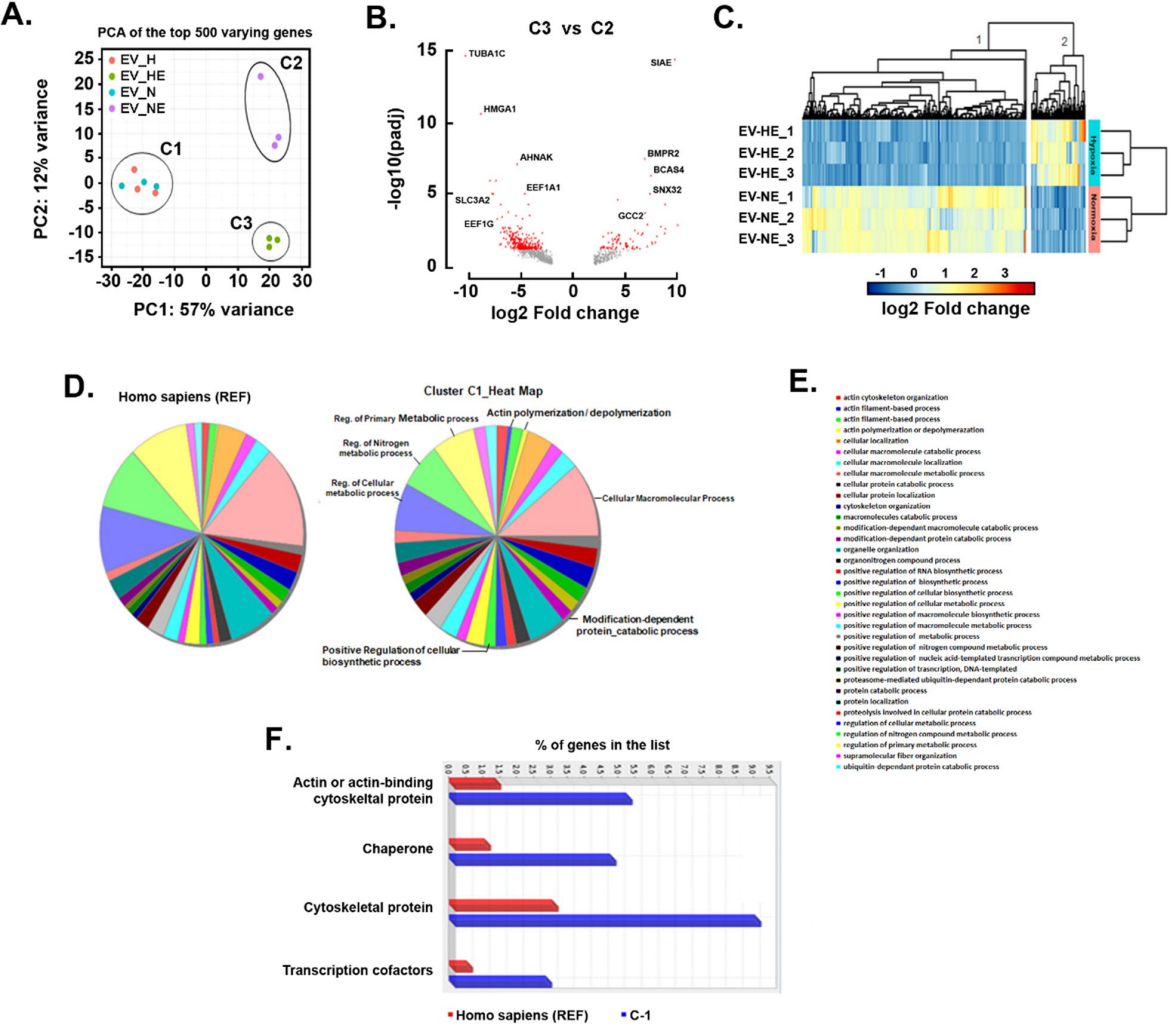
*Transcriptomic analysis of the influence of low oxygen tension and EGCG in loading the MDA-MB-231-derived EVs*. **A** Principal Component Analysis (PCA) of the top 500 differential expressed genes (DEG) identified in the samples. **B** Venn diagram showing the number of DEG detected with a log2 FC ≥|2| in the EVs obtained by adding 30 µM EGCG at different oxygen tensions. The analysis was performed by comparing the EGCG-EVs obtained in hypoxia (EV_HE, C3) vs EGCG-EVs obtained in normoxia (EV_NE, C2). Genes with a significant or non-significant value were coloured in red and grey respectively. **C)** Robust *k*-means clustering visualized as a heatmap of the individual samples and their DEG with a log2 FC ≥|2| and *p*-adjusted value < 0.05. **D** Gene ontology (GO)-SLIM PANTHER analysis showing the biological process that involves genes downregulated by HE (cluster 1, heat map). **E** List of all biological processes downregulated by HE**. F** GO-SLIM PANTHER analysis of the downregulated genes in cluster 1 of the heatmap, showing their protein class. Fisher’s exact test and the false discovery rate (FDR) correction were used during the GO-SLIM analysis

We next questioned whether EVs content is altered in conditions which mimic the patho-physiological conditions of a solid tumor microenvironment, where nutrients are limited and oxygen tension is low. Given that such starvation and hypoxic conditions have a direct impact on cell metabolism, we assessed the mRNA cargo of the EVs derived from MDA-MB-231 cells and whether EGCG additionally altered such content. EVs and EGCG-EVs were isolated from normoxic (21% O_2_) and hypoxic (1% O_2_) culture conditions, and total RNA extracted for RNA-Seq analysis. Principal component analysis (PCA) shows that EVs samples obtained from cells cultured in normoxia (EV_N) or hypoxia (EV_H) are very similar (cluster 1, C1) (Fig. 7A). Comparing the transcript content of EV_H and EV_N (C1, Fig. 7A), only nine DEG were identified (fold change FC >|2| and adjusted *p*-value < 0.05) (Table.2). Among those genes, five were upregulated and were directly involved in the hypoxia-mediated process. These included cell death (*FAM162A*), mitochondria function (*ISCA1*), metabolism (*PM20D2*, *SLC2A3*), and angiogenesis (*ADM*). The four downregulated genes were associated with gene expression machinery (*NIP7*, *MRPS7*, *MT-TP*, *ZNF585A*).

**Table 2:** *Genes modulated in hypoxia vs normoxia with a p-adjusted value* < *0.05 and a log2 FC* > *l2l*

Gene Ensemble ID	log2 FC	*p*-adjusted value	GenCards annotations
PM20D2 ENSG00000146281,6	4.27	0.032	*Peptidase M20 Domain Containing 2*. Hydrolase activity. Function in metabolite repair mechanism
FAM162A ENSG00000114023,15	3.52	0.001	*Family With Sequence Similarity 162 Member A*. Hypoxia-induced cell death, the release of cytochrome C, caspase activation (CASP9) and inducing mitochondrial permeability transition
ISCA1 ENSG00000135070,15	2.60	0.049	*Iron-Sulfur Cluster Assembly 1*. Mitochondrial protein. Function in electron-transfer reactions
ADM ENSG00000148926,10	2.23	0.001	*Adrenomedullin*. Functions in vasodilation, hormone secretion regulation, angiogenesis promotion, and antimicrobial activity
SLC2A3 ENSG00000059804,16	2.09	0.001	*GLUT3*. Glucose and other monosaccharides transporter
NIP7 ENSG00000132603,15	− 2.20	0.049	*Nucleolar Pre-rRNA Processing Protein NIP7*. RNA binding
MRPS7 ENSG00000125445,11	− 2.41	0.036	*Mitochondrial Ribosomal Protein S7*. RNA binding and structural constituent of ribosome
MT-TP ENSG00000210196,2	− 2.69	0.032	*Mitochondrially Encoded TRNA-Pro (CCN*). Associated with the tRNA class
ZNF585A ENSG00000196967,11	− 3.97	0.048	*Zinc Finger Protein 585A*. Function as a transcription factor

Interestingly, while oxygen levels had no significant impact on the transcript content of vesicles produced by untreated cells, the addition of EGCG drives the production of vesicles with distinctive transcript signatures linked to oxygen levels (Fig. 7A, C2 and C3 clusters). This suggests that hypoxic conditions potentiate the action of EGCG. The analysis of the transcripts isolated in EV_HE (C3) vs EV_NE (C2) resulted in the detection of 2,640 gene products, from which 553 were differentially regulated between the experimental conditions (Table 3).

**Table 3:** DESeq results obtained from the comparison of the EV_HE vs EV_NE

*p*-adjusted value	Fold change	Number of genes
Significant	Upregulated	107
Significant	Downregulated	446
Not significant	–	2087

The Volcano Plot in Fig. 7B depicts the behaviour of the DEG with an absolute fold change greater than two-fold and colored according to their adjusted *p*-value (red or grey for DEG statistically significant or not respectively). Addition of EGCG in hypoxic cell culture conditions showcases a noticeable gene downregulation (446 downregulated genes vs. 107 upregulated genes). Among the most upregulated genes with the highest statistical differences were the sialic acid acetylesterase enzyme (*SIAE*), the bone morphogenetic protein receptor type 2 (*BMPR2*), the oncogene breast carcinoma amplified sequence 4 (*BCAS4*), genes associated with the vesicular transport like the sorting nexin 32 (*SNX32*), and the GRIP and coiled-coil domain containing 2 (*GCC2*). On the opposite, among those genes with the highest downregulation, we found the cytoskeleton constituent tubulin alpha 1c (*TUBA1C*), the high mobility group AT-Hook 1 (*HMGA1*) associated to gene transcription, the scaffolding protein neuroblast differentiation-associated (*AHNAK*), the coding genes associated to ribosomal functionality the eukaryotic translation elongation factors 1 alpha 1 and 1 gamma (*EEF1A1* and *EEF1G*), and the transmembrane protein encoded gene solute carrier family 3 member 2 (*SLC3A2*).

Next, we performed a *k*-means clustering analysis with the 553 DEG and generated the heat map containing each condition replicate (Fig. 7C). As expected, we detected two major clusters comprising 107 genes, and the other one with the remaining 446 genes. A detailed information of these DEG is provided in a supplementary EXCEL data sheet (table is provided as Additional file 5). Unfortunately, no association was found upon Gene ontology (GO) enrichment analysis performed (FDR < 0.05) for the upregulated genes (cluster 2, Fig. 7C). Nevertheless, most of the upregulated genes coded for proteins involved in the regulation of gene expression, mitochondria components and protein trafficking and recycling. Importantly, there were genes upregulated within the EGCG-EVs which associated with the inflammatory response (*HAVCR2*, *LRRFIP2*), cell proliferation (*CCPG1*, *EXOSC2*), apoptosis (*BBC3*) and oncogenesis (*ZMAT3*, *ZNF124*, *ErbB-3*, *SH3D19*, *REL*). When the same analysis was performed with the genes downregulated in EV_HE (cluster 1, Fig. 7C), we identified genes that were tribute to biological processes associated with regulation of cell metabolism, biosynthesis, and cell mobility like the polymerization/depolymerization of actin (Fig. 7D). Furthermore, in comparison with the EV_NE, the EV_HE vesicles had less genes coding for cytoskeletal proteins, chaperons, and transcription factors (Fig. 7E).

## Discussion

The release of small EVs loaded with bioactive macromolecules is an efficient communication and paracrine regulation mechanism linking the tumor niche to its neighbouring cells. Cancer cells attract and trigger dedifferentiation of neighbouring cells to acquire a pro-tumoral phenotype as described for the TAM [50], cancer-associated fibroblasts (CAF) [51], and cancer-associated adipocytes (CAA) [52]. In our previous study, we demonstrated that the secretome of a TNBC cell line (MDA-MB-231) induced the CAA-like phenotype in hADMSC, a process inhibited by EGCG [27]. To decipher the different components within that secretome, the present study focused on the role of the tumor-derived EVs and their paracrine regulation over the hADMSC, as well as the effect of EGCG on the vesicle’s load and biological effect.

EVs have been demonstrated to regulate processes like inflammation and tissue repair, as well as to condition the premetastatic niche [10]. EVs can travel to distant sites and transfer biological information to the recipient cells [4, 9, 12]. This can be achieved through direct fusion of the vesicles to the plasma membrane via receptor-ligand interactions, membrane fusion or by releasing their load within the cytosol [53]. Our samples were enriched in exosomes according to the isolation method used and the detection of exosomal markers, but the presence of vesicles with different size cannot be dismissed. With regards to such size distribution, both EVs and EGCG-EVs vesicles behaved similarly. In addition to its antioxidant properties, pro-oxidant properties have also been reported for EGCG both in vitro and in vivo [54, 55]. How this could directly affect the membrane composition of the vesicles, as well as the response of the targeted cells, remains to be addressed. However, in our experimental conditions no differences were observed in terms of MFI in the labelled vesicles (Additional file 6: Fig. S2D) or within the recipient cells (Additional file 6: Fig. S1C) implying that the staining and interaction/fusion capacity of the vesicles remained unaltered.

Here, we demonstrate that TNBC cell-derived EVs can interact with hADMSC and induce a pro-migratory and pro-inflammatory phenotype. Accordingly, the inflammation-associated IL-17 pathway was enriched during the transcriptomic analysis of the EVs cargo. Next, we validated the regulation of other inflammatory markers as EVs induced several of the genes involved in this signaling cascade including *IL-1B, CXCL1* and *CXCL2*, *CCL2*, *CCL7* and *CCL20* [56], while EGCG-EVs reduced most of them. We highlighted the modulation of the CAA markers by the vesicles suggesting that this phenotype can be induced in the hADMSC. Similar observations were found in adipocytes co-cultured with exosomes derived from hepatocellular carcinoma, where the induction of pro-inflammatory cytokines IL-6, IL-8, IL-1β and CCL2 promoted tumor growth and angiogenesis [57]. Interestingly, EGCG-EVs specifically induced IL-6 along with the chemokines *CXCL1*, *CXCL3* and *CXCL8*. These cytokines are key regulators of the acute response during inflammation and immune response and can also contribute to tissue homeostasis by inducing the recruitment of innate immune system cells [58, 59].

By interacting with hADMSC, both EVs preparations (EVs and EGCG-EVs) specifically triggered the DNA damage response pathway CHK-2 and the stress-activated protein kinase c-Jun/JNK pathway. CHK-2 is activated in response to the oxidative stress generated upon nutrient deprivation and maintains the reactive oxygen species (ROS) homeostasis [60]. Hence, there appears to be some stress-mediated effect of the EVs samples over the cells, in addition to the nutrient deprivation. In opposition to the EVs-mediated effect, the EGCG-EVs failed to activate the AKT pathway and reduced the GSK3β pathway activation status. AKT activation has a positive regulation over several processes including metabolism, proliferation, and cell survival [61]. On the other hand, GSK3β relays a signal transduction cascade involved in cellular processes like gene transcription, cell proliferation, and apoptosis [62]. This protein can be phosphorylated on different serine residues by other kinases including AKT [63] and p38 [64], correlating with the inhibition of its kinase activity, or by MEK1/2 leading to its induction [65]. The role of AKT mediating the activation of the c-Jun/caspase-3 axis in response to the endoplasmic reticulum stress, which renders in attenuation of the P21 expression level in prostate cancer cells model was recently documented [66]. Then, cells exited from a senescence state to enter apoptosis-mediated cell death. Here, the EGCG-EVs genetic content altered pathways associated to ROS, cell cycle, cellular senescence, HIF-1α and Notch. The latter has been also associated with the induction of AKT signalling pathway and P21 expression in T-cell lymphoblastic leukemia [67]. Besides, we detected a sustained activation of the P38/MAPK pathways which corresponds with the induction of senescence in response to chronic DNA-damage [68]. These results may suggest that EGCG-EVs can rescue cells from senescence since its capacity to reduce the P21 and β-gal expression was demonstrated. In our study, SOD2 was the only senescence marker induced explicitly by the EGCG-EVs, a fact previously reported for EGCG during an in vivo study and linked to its capacity to prevent the oxidative stress caused by free fatty acid-induced insulin resistance [69].

The pro-tumoral role of senescent cells has been described as promoting low-grade inflammation [31] and the CCL2-mediated recruitment of myeloid and NK cells [70]. Also, senescent tumor cells have been reported to have increased migration capacity and to be often present at the tumor invasive front [71]. Here, the EVs increased the expression of pro-inflammatory and senescence markers in hADMSC, as well as their migration rate. These shreds of evidence highlight the predominant inhibitory effect of EGCG-EVs in both biological processes. Whereas these vesicles triggered IL-6 and CXCL8 expression, further studies will be required to clarify how such induction may impact tumor progression.

In addition, we detected the presence of mtDNA and functional mitochondrial content within the isolated vesicles, which would be potentially transferred to the recipient cells. It has been reported that mitochondria and their components, when transferred to recipient cells, induce an inflammatory response [18, 19], and increase invasiveness in tumor cells [72, 73]. Previous studies have shown that intercellular transfer of functional mitochondrial content can rescue injured or UV-treated prostate cancer cells (PC12) [22, 74]. Since the integrity of the extracellular mitochondrial content is affected by the EGCG treatment, the bioenergetic state of the recipient cells could be significantly modulated. However, the nature of the factors transmitted varies according to the cell culture conditions and cell line models used [75]. Our findings support previous reports since mitophagy was detected among the pathways enriched during our transcriptomic analysis of the EGCG-EVs, and this was confirmed later by the reduction in the mitochondrial content within these vesicles. Whether this correlates with the anti-inflammatory properties of the EGCG-EVs has yet to be established.

The influence of culture conditions in the loading selection of the EVs content has also been extensively studied for the micro-RNA (miRNA) profile [76]. In the present study, we focused on the sorting of the RNA content that can be transcribed and translated into proteins. Considering the impact of hypoxia on cell metabolism and secretory profiles, we expanded our analysis on how low oxygen tension influences the transcriptomic profile within the vesicles in combination with EGCG. Contrary to what has been published [77], in our experimental condition, hypoxia per se did not impact the transcripts levels sorted into the vesicles. However, when cells were concomitantly cultured in EGCG, two different EVs clusters were identified (Fig. 7A). Overall, the combined effects of hypoxia and EGCG (EV_HE) caused the downregulation of most genes identified in the EV_NE. The main protein classes that were downregulated in the EV_HE were associated with cytoskeleton and actin polymerization/depolymerization processes that are involved in exosome biogenesis and secretion mechanisms [78]. Transcription factors and chaperons were also identified, suggesting their involvement in the regulation of metabolic and biosynthetic cellular processes. In general, the combination of hypoxia and EGCG appeared to attenuate the paracrine regulatory effect of the vesicles obtained without the stress of low oxygen tension. However, we observed that the EV_HE were enriched in genes associated with inflammation, cell survival and oncogenesis. This further highlights the importance of mimicking in vitro as closely as possible the tumor microenvironment through cell culture conditions in studying EVs paracrine regulation mechanisms.

## Conclusions

The present proof of concept study reveals that EGCG can alter the sorted genetic material found within the TNBC cells-derived EVs, and this will require to be further explored in other breast cancer cell models. As evidenced, EGCG reduced the capacity of the EVs to trigger the expression of pro-inflammatory and senescence markers that, otherwise, could contribute to the acquisition of a chemoresistance phenotype. How gene expression changes translate into functional consequences upon EGCG treatment, in combination with current chemotherapeutic approaches, should be investigated in further studies to confirm the potential chemoresistance phenotype induced by the horizontal transfer of mitochondrial content. This evidence was further supported by its inhibitory effects over crucial pathways involved in cell proliferation and cell death including, in part, GSK3β and Akt. Of importance, EGCG caused a reduction in the mitochondrial content of cancer cells-derived EVs, reinforcing its overall antitumoral role. Circulating diet-derived polyphenols may therefore represent an efficient chemopreventive strategy to reduce the paracrine regulation that TNBC cells exert within their surrounding adipose tissue environment.

### Supplementary Information

**Additional file 1.** RNA-Seq analysis of differentially expressed genes (DEG) between EVs isolated from control (EVs) or EGCG-treated cells (EGCG-EVs).

**Additional file 2.** 3D animated field 1 of mitochondrial delivery (red staining) within hADMSC.

**Additional file 3.** 3D animated field 2 of mitochondrial delivery (red staining) within hADMSC.

**Additional file 4.** 3D animated field 3 of mitochondrial delivery (red staining) within hADMSC.

**Additional file 5.** Analysis of the 553 DEG and of the two major clusters identified.

**Additional file 6: Figure S1.** *Quantification by flow cytometry of the EVs samples*. EVs and EGCG-EVs were isolated, and 20 µL of the samples were stained with 100 nM MemGlow followed by flow cytometry analysis. **A** Gating strategy and definition of the unstained population. **B** Representative quantification of a batch of EVs (N170622). **C** Representative quantification of a batch of EGCG-EVs (NE170623). Highlighted in red are the P2-positive population with the number of vesicles counted in an acquisition volume of 30 µL. **D** Paired experimental means of each EVs batch's mean fluorescence intensity (MFI).** E** Paired experimental counting of the number of particles. Wilcoxon-matched pairs signed rank test was used to establish significant statistical differences. **Figure S2.** *Comparing the fusion capacity of MemGlow-stained EVs*. EVs and EGCG-EVs were isolated, labelled with 100 nM MemGlow, washed by ultracentrifugation (1 hour at 100,000g) and resuspended in basal media (BM). Next, hADMSC (10,000 cells/condition) were incubated in suspension with the vesicles at a ratio Cells:EVs of 1:1, for 1 hour at 37 °C and 5% CO_2_ atmosphere. Flow cytometry determination of the number of FL-1-positive cells. **A** Gating strategy used for samples incubated with BM as a negative control. **B** Representative plotting of the MemGlow-488-positive cells resulting from the co-incubation with EVs or EGCG-EVs. **C** Representative plots of the mean of fluorescence intensity (MFI) of the hADMSC incubated with BM (black line), EVs (aqua-coloured line) or with EGCG-EVs (dark blue line). **Figure S3.** *Evaluating the effect of EGCG over the mitoTracker dye*. MDA-MB-231 cells were seeded in a 6-well plate, incubated with mitoTracker Deep Red (MT), resuspended in negative media (NM), and added at a final concentration of 200 nM. After washing, the cells were kept for 24 hours in negative media (NM) or NM+EGCG at 10 or 30 µM, respectively. Then, cells were analyzed by flow cytometry. **A** A representative dot plot of unstained cells (negative control, MT-), cells stained with MT and maintained in NM (positive control, MT+), cells stained and incubated with 10 µM (MT+ EGCG-10) or with 30 µM (MT+ EGCG-30). **B **Bar graph of the mean of the fluorescence intensity (MFI) of the positive population (n=2). **Figure S4.** *Mitochondria components present within MDA-MB-231-derived EVs can be transferred into hADMSC*. MDA-MB-231 cells were seeded in 175 cm flasks, incubated with mitoTracker Deep Red (MTR), resuspended in negative media (NM), and added at a final concentration of 200 nM. After washing, the cells were kept for 24 h in negative media (NM). EVs were isolated as described in the Methods section and protected from light. hADMSC were then seeded ontop of tissue culture glass slides (polystyrene 4 cambers, REF 354114, Falcon, NY) coated with Poly-lysine. 200 μl of MTR+EVs were then resuspended in NM, incubated for 4 hours. Cells were then labelled with 100 nM MemGlow, incubated for 20 minutes at RT and in the dark, fixed in 1% paraformaldehyde sol at 2%. Dapi was added to stain the nucleus and pictures taken using a fluorescence microscope. Red staining is representative of mitochondrial material delivered within hADMSC (stained in green). Animated 3D GIF files of these respective fields are also provided as Supplemental material (Additional file 2_Field_1; Additional file 3_Field_2; Additional file 4_Field_3).

## Data Availability

All material will be made available from the corresponding author upon reasonable request, and data generated or analyzed during this study are included in this published article.

## Abbreviations

hADMSC: Human adipocyte-derived mesenchymal stem cells
BSA: Bovine serum albumin
CAA: Cancer-associated adipocyte
CAF: Cancer-associated fibroblast
DEG: Differentially expressed genes
DLS: Dynamic light scattering
EGCG: Epigallocatechin-3-gallate
EGFR: Epidermal growth factor receptor
EVs: Extracellular vesicles
FBS: Fetal bovine serum
FC: Fold change
FDR: False discovery rate
GO: Gene ontology
GSEA: Gene set enrichment analysis
miRNA: MicroRNA
mitoMPs: CD44^+^/MTR^+^ microparticles
MPs: CD44^+^/MTR^−^ microparticles
mtDNA: Mitochondrial DNA
MTR: MitoTracker Deep Red
PCA: Principal component analysis
ROS: Reactive oxygen species
SDS: Sodium dodecyl sulfate
SEM: Standard error of the mean
TAM: Tumor associated macrophage
TNBC: Triple-negative breast cancer
