# Supplementary figures and images for: EGCG inhibits the inflammation and senescence inducing properties of MDA-MB-231 triple-negative breast cancer (TNBC) cells-derived extracellular vesicles in human adipose-derived mesenchymal stem cells
Source: Cancer Cell Int. 2023 Oct 13;23:240. doi: 10.1186/s12935-023-03087-2 (PMC10576371; doi:10.1186/s12935-023-03087-2)

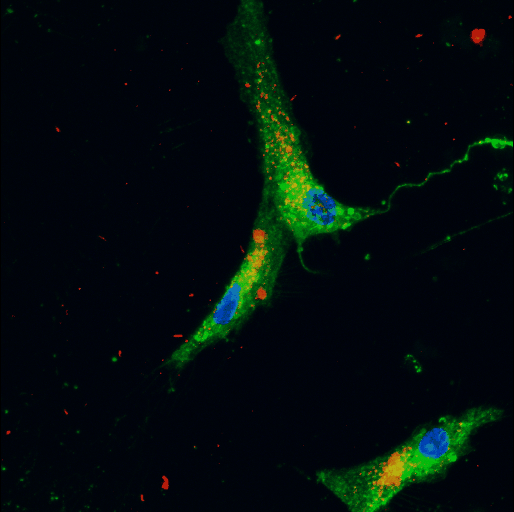

Supplement: Supplementary file 2 — Additional file 2. 3D animated field 1 of mitochondrial delivery (red staining) within hADMSC. [file 12935_2023_3087_MOESM2_ESM.gif]

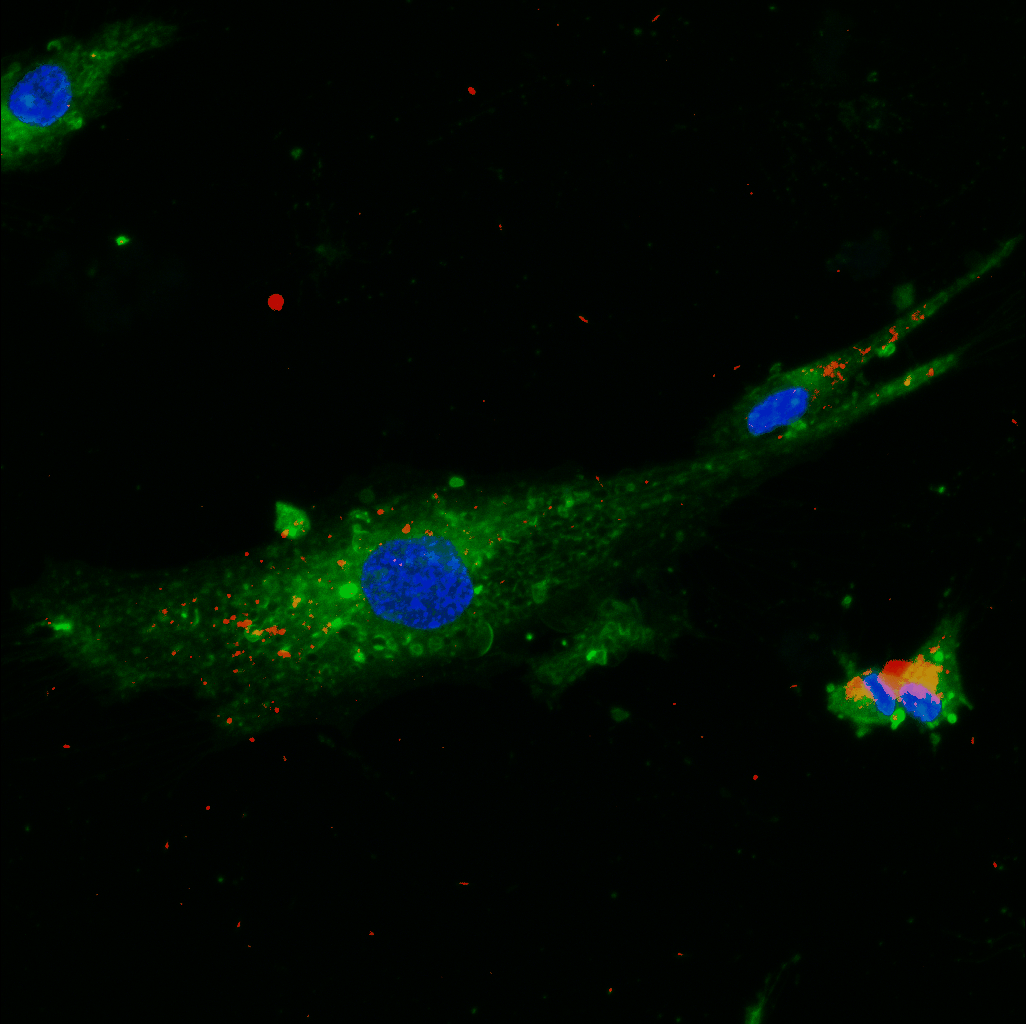

Supplement: Supplementary file 3 — Additional file 3. 3D animated field 2 of mitochondrial delivery (red staining) within hADMSC. [file 12935_2023_3087_MOESM3_ESM.gif]

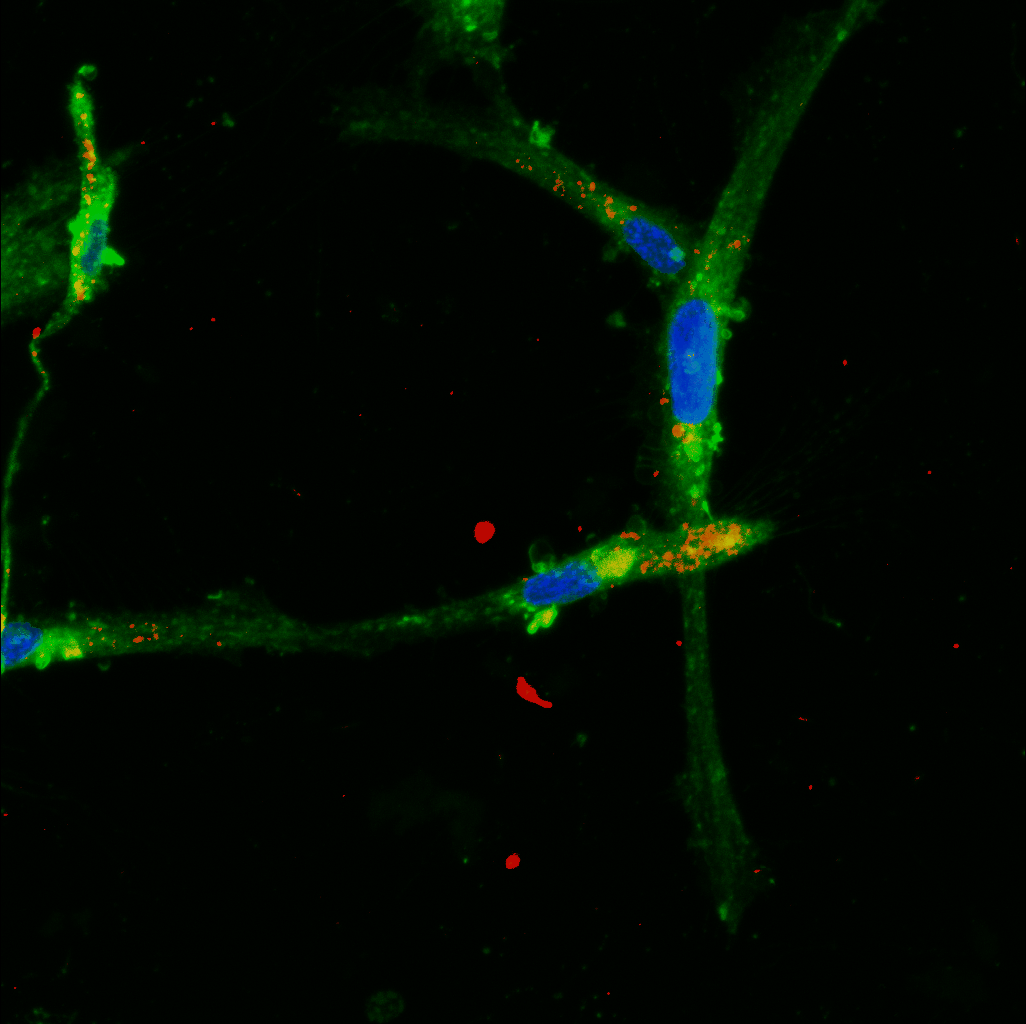

Supplement: Supplementary file 4 — Additional file 4. 3D animated field 3 of mitochondrial delivery (red staining) within hADMSC. [file 12935_2023_3087_MOESM4_ESM.gif]
