# Supplementary material for: EGCG inhibits the inflammation and senescence inducing properties of MDA-MB-231 triple-negative breast cancer (TNBC) cells-derived extracellular vesicles in human adipose-derived mesenchymal stem cells
Source: Cancer Cell Int. 2023 Oct 13;23:240. doi: 10.1186/s12935-023-03087-2 (PMC10576371; doi:10.1186/s12935-023-03087-2)
Supplement: Supplementary file 6 — Additional file 6: Figure S1. Quantification by flow cytometry of the EVs samples. EVs and EGCG-EVs were isolated, and 20 µL of the samples were stained with 100 nM MemGlow followed by flow cytometry analysis. A Gating strategy and definition of the unstained population. B Representative quantification of a batch of EVs (N170622). C Representative quantification of a batch of EGCG-EVs (NE170623). Highlighted in red are the P2-positive population with the number of vesicles counted in an acquisition volume of 30 µL. D Paired experimental means of each EVs batch's mean fluorescence intensity (MFI). E Paired experimental counting of the number of particles. Wilcoxon-matched pairs signed rank test was used to establish significant statistical differences. Figure S2. Comparing the fusion capacity of MemGlow-stained EVs. EVs and EGCG-EVs were isolated, labelled with 100 nM MemGlow, washed by ultracentrifugation (1 hour at 100,000g) and resuspended in basal media (BM). Next, hADMSC (10,000 cells/condition) were incubated in suspension with the vesicles at a ratio Cells:EVs of 1:1, for 1 hour at 37 °C and 5% CO2 atmosphere. Flow cytometry determination of the number of FL-1-positive cells. A Gating strategy used for samples incubated with BM as a negative control. B Representative plotting of the MemGlow-488-positive cells resulting from the co-incubation with EVs or EGCG-EVs. C Representative plots of the mean of fluorescence intensity (MFI) of the hADMSC incubated with BM (black line), EVs (aqua-coloured line) or with EGCG-EVs (dark blue line). Figure S3. Evaluating the effect of EGCG over the mitoTracker dye. MDA-MB-231 cells were seeded in a 6-well plate, incubated with mitoTracker Deep Red (MT), resuspended in negative media (NM), and added at a final concentration of 200 nM. After washing, the cells were kept for 24 hours in negative media (NM) or NM+EGCG at 10 or 30 µM, respectively. Then, cells were analyzed by flow cytometry. A A representative dot plot of unstaine [file 12935_2023_3087_MOESM6_ESM.docx]

**SUPPLEMENTARY FIGURES**

**Supplementary Figure S1**

**Figure S1:** *Quantification by flow cytometry of the EVs samples*. EVs and EGCG-EVs were isolated, and 20 µL of the samples were stained with 100 nM MemGlow followed by flow cytometry analysis. **A)** Gating strategy and definition of the unstained population. **B)** Representative quantification of a batch of EVs (N170622). **C)** Representative quantification of a batch of EGCG-EVs (NE170623). Highlighted in red are the P2-positive population with the number of vesicles counted in an acquisition volume of 30 µL. **D)** Paired experimental means of each EVs batch's mean fluorescence intensity (MFI). **E)** Paired experimental counting of the number of particles. Wilcoxon-matched pairs signed rank test was used to establish significant statistical differences.

**Supplementary Figure S2**

**Figure S2:** *Comparing the fusion capacity of MemGlow-stained EVs*. EVs and EGCG-EVs were isolated, labelled with 100 nM MemGlow, washed by ultracentrifugation (1 hour at 100,000g) and resuspended in basal media (BM). Next, hADMSC (10,000 cells/condition) were incubated in suspension with the vesicles at a ratio Cells:EVs of 1:1, for 1 hour at 37°C and 5% CO_2_ atmosphere. Flow cytometry determination of the number of FL-1-positive cells. **A)** Gating strategy used for samples incubated with BM as a negative control. **B)** Representative plotting of the MemGlow-488-positive cells resulting from the co-incubation with EVs or EGCG-EVs. **C)** Representative plots of the mean of fluorescence intensity (MFI) of the hADMSC incubated with BM (black line), EVs (aqua-coloured line) or with EGCG-EVs (dark blue line).

**Supplementary Figure S3**

**Figure S3:** *Evaluating the effect of EGCG over the mitoTracker dye*. MDA-MB-231 cells were seeded in a 6-well plate, incubated with mitoTracker Deep Red (MT), resuspended in negative media (NM), and added at a final concentration of 200 nM. After washing, the cells were kept for 24 hours in negative media (NM) or NM+EGCG at 10 or 30 µM, respectively. Then, cells were analyzed by flow cytometry. **A)** A representative dot plot of unstained cells (negative control, MT-), cells stained with MT and maintained in NM (positive control, MT+), cells stained and incubated with 10 µM (MT+ EGCG-10) or with 30 µM (MT+ EGCG-30). **B)** Bar graph of the mean of the fluorescence intensity (MFI) of the positive population (n=2).

**Supplementary Figure S4**

**Figure S4:** *Mitochondria components present within MDA-MB-231-derived EVs can be transferred into hADMSC*. MDA-MB-231 cells were seeded in 175 cm flasks, incubated with mitoTracker Deep Red (MTR), resuspended in negative media (NM), and added at a final concentration of 200 nM. After washing, the cells were kept for 24 hours in negative media (NM). EVs were isolated as described in the Methods section and protected from light. hADMSC were then seeded ontop of tissue culture glass slides (polystyrene 4 cambers, REF 354114, Falcon, NY) coated with Poly-lysine. 200 μl of MTR+EVs were then resuspended in NM, incubated for 4 hours. Cells were then labelled with 100 nM MemGlow, incubated for 20 minutes at RT and in the dark, fixed in 1% paraformaldehyde sol at 2%. Dapi was added to stain the nucleus and pictures taken using a fluorescence microscope. Red staining is representative of mitochondrial material delivered within hADMSC (stained in green). Animated 3D GIF files of these respective fields are also provided as Supplemental material (Additional file 2_Field_1; Additional file 3_Field_2; Additional file 4_Field_3).
